# Identification of SLC7A1 as a potential therapeutic target for high-grade meningioma

**DOI:** 10.1038/s41420-025-02783-4

**Published:** 2025-11-03

**Authors:** Lairong Song, Xiaojie Li, Da Li, Kaibing Tian, Ke Wang, Junting Zhang, Liang Wang, Zhen Wu

**Affiliations:** 1 Department of Neurosurgery, Beijing Tiantan Hospital, Capital Medical University, Beijing, 100071 China; 2 Beijing Neurosurgical Institute, Capital Medical University, Beijing, 100071 China; 3 China National Clinical Research Center for Neurological Diseases, Beijing, 100071 China

**Keywords:** Targeted therapies, Target identification

## Abstract

High-grade meningioma remains a therapeutic challenge. The first-line guideline drugs for high-grade meningioma are still lacking, highlighting the urgent need to uncover new therapeutic targets. As a cationic amino acid transporter, SLC7A1 was highly expressed in high-grade meningioma and associated with poor prognosis of patients. In this study, transcriptomic analyses at both the single-cell and bulk levels were employed to investigate the molecular function of SLC7A1. The Genomics of Drug Sensitivity in Cancer (GDSC) database was utilized for predicting potential drugs targeting high-SLC7A1 meningiomas. RNA sequencing was conducted to explore the differential activity of cancer hallmark pathways and transcription factors. The effects of SLC7A1 knockdown and drug treatment were validated in vitro and in vivo. Our results revealed that SLC7A1 regulates multiple signaling pathways involved in tumor proliferation, including E2F targets, G2M checkpoint, and MYC targets. Knockdown of SLC7A1 significantly inhibited the proliferation, invasion, and xenograft tumor growth of meningioma cells. Furthermore, SLC7A1-FOXM1/E2F4 regulatory axis may contribute to the malignant progression of meningioma. AZ628, predicted as a small molecule drug targeting high-SLC7A1 meningiomas, exhibited an excellent antitumor effect against meningioma in vitro, in vivo, and in organoid models. Additionally, AZ628 treatment also inhibited the transcriptional activity and protein expression of FOXM1 and E2F4, mirroring the effects of SLC7A1 knockdown in meningioma. In brief, our study demonstrated the tumor-promoting function of SLC7A1 by regulating the transcription factors FOXM1 and E2F4 in meningioma and identified SLC7A1 as a potential therapeutic target. Meanwhile, AZ628 is a promising small molecule drug for high-grade meningioma.

## Introduction

Meningioma is the most common intracranial tumor in adulthood. Although most meningiomas are benign, ~20% exhibit malignant biological features and are categorized as high-grade (WHO grade 2 or 3) [1]. Benign meningioma usually has a favorable outcome following surgical resection. In contrast, high-grade meningioma is associated with a higher recurrent rate and worse prognosis, even after surgery and radiotherapy [2]. High-grade meningioma remains a therapeutic challenge to neurosurgeons and oncologists. Conventional radiotherapy is the only effective adjuvant treatment that can delay meningioma recurrence [3]. Despite extensive efforts, chemotherapeutic agents have demonstrated limited efficacy in treating high-grade meningioma [3]. Hence, there is an urgent demand to identify potential molecular targets for the effective treatment.

Amino acid metabolism reprogramming plays a critical role in the development and progression of tumors [4]. Tumor cells often undergo remodeling of amino acid uptake and amino acid metabolism processes to meet their own demands [4]. Due to the hydrophilic nature of amino acids, they cannot be absorbed and utilized by tumor cells through free diffusion [4]. Therefore, various amino acid transporters are required to assist the uptake and balance of amino acid [4]. For example, the amino acid transport SLC7A5 plays a critical role in colorectal tumorigenesis by maintaining intracellular amino acid levels [5]. Urea cycle disorder is common in tumor cells, which leads to defective arginine synthesis, also known as arginine auxotrophy [6, 7]. The survival of arginine-auxotrophic tumor cells is highly dependent on extracellular arginine uptake [6, 8]. The arginine transporter SLC7A3 has been proved to promote the epithelial-mesenchymal transition and metastasis of osteosarcoma through increasing the uptake of arginine [9]. The arginine transporter SLC7A1 has been demonstrated to be upregulated in various tumors including liver cancer, ovarian cancer, breast cancer, leukemia, and exhibits pro-oncogenic functions [7, 10–12]. Considering the crucial role of arginine in tumor, arginine depletion therapy is regarded as a promising anti-tumor strategy. Pegylated recombinant mutant human arginase I (BCT-100), as a synthetically engineered arginine-degrading enzyme, has been undergoing clinical trials for various malignancies such as liver cancer, leukemia [13].

However, arginine depletion therapy is a double-edged sword. Although it can partially inhibit the proliferation of tumor cells by depleting arginine, it can also impair the activation and functionality of T cells within the tumor microenvironment [14, 15]. Therefore, targeting the arginine transporters on tumor cells to inhibit their arginine uptake may represent a more promising direction. SLC7A1, also referred to as cationic amino acid transporter-1 (CAT-1), is a membrane transporter responsible for the uptake of extracellular arginine [16]. Knockdown of SLC7A1 could significantly inhibit the uptake of arginine in tumor cells [7, 10, 11]. Although several studies have suggested the oncogenic role of SLC7A1 in various tumors, the downstream pathways and molecular targets regulated by SLC7A1, as well as its functional role in meningioma, remain largely unexplored.

Here, we revealed that SLC7A1 was upregulated in high-grade meningioma, and high SLC7A1 expression was associated with poor prognosis of meningioma patients. Furthermore, we conducted a comprehensive analysis of the role of SLC7A1 in meningioma at both the single-cell and bulk levels. Experimental validation was also performed accordingly. This study would provide new insight into the molecular-targeted therapy of high-grade meningioma.

## Results

### Single-cell analysis reveals that SLC7A1 is closely associated with the proliferation pathways in meningioma cells

**Fig. 1:**
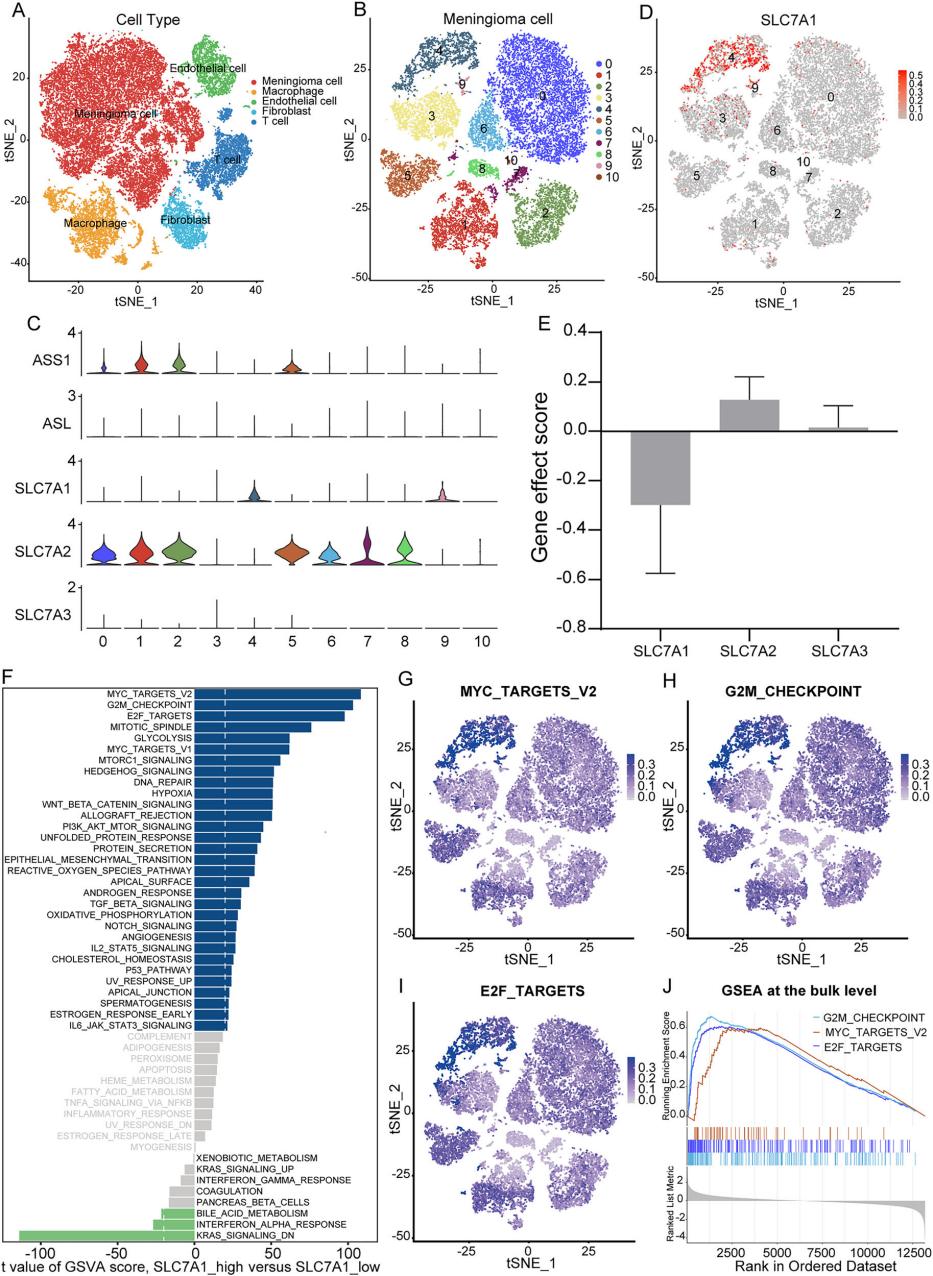
GSVA at the single-cell level reveals SLC7A1-related biological processes. **A** tSNE plot of 32714 cells with each cell color-coded by cell type. **B** tSNE plots of 19429 meningioma cells with each cell color-coded by cell cluster. **C** Violin plot displaying the expression levels of ASS1, ASL, SLC7A1, SLC7A2, and SLC7A3 in different clusters of meningioma cells. **D** tSNE plots of 19429 meningioma cells with each cell color-coded by the expression level of SLC7A1. **E** The effect of CRISPR knockout of SLC7A1, SLC7A2, and SLC7A3 in pan-cancer from the DEPMAP portal. **F** Differences in pathway activities scored per cell by GSVA between high- and low-SLC7A1 meningioma cell clusters. **G**–**I** tSNE plots of 19429 meningioma cells with each cell color-coded by GSVA score of the MYC targets, G2M checkpoint, and E2F targets pathways. **J** GSEA at the bulk level displaying the activation of MYC targets, G2M checkpoint, and E2F targets pathways in the high-SLC7A1 group of the GSE136661 dataset.

The scRNA-seq data of six meningioma samples were analyzed. A total of 32,714 cells were divided into five major clusters, including meningioma cell, macrophage, endothelial cell, fibroblast, and T cell (Fig. 1A). Subsequently, 19,429 meningioma cells were selected and then divided into 11 minor clusters (Fig. 1B). Argininosuccinate synthetase 1 (ASS1) and argininosuccinate lyase (ASL) are two key enzymes in the de novo synthesis of arginine [4]. We analyzed the expression of ASS1 and ASL in 11 clusters of meningioma cells. The results showed that several clusters of meningioma cells expressed ASS1, while nearly all meningioma cell clusters exhibited negligible expression of ASL (Fig. 1C). This suggests the presence of urea cycle disorder and arginine auxotrophy in meningioma. The survival of meningioma cells may highly rely on extracellular arginine uptake.

As members of the cationic amino acid transport (CAT) family, SLC7A1, SLC7A2, and SLC7A3 are responsible for the majority of cellular arginine uptake [4]. Single-cell analysis revealed that SLC7A1 and SLC7A2 are expressed in meningioma cells, exhibiting mutual exclusivity in expression, while SLC7A3 is barely expressed (Fig. 1C). Furthermore, we analyzed the functional roles of SLC7A1, SLC7A2, and SLC7A3 in pan-cancer using the CRISPR knockout dataset from the DEPMAP portal. The results showed that SLC7A1 displayed significantly lower gene effect score compared to SLC7A2 and SLC7A3 (Fig. 1E, Supplementary Fig. S2), suggesting that SLC7A1 plays a more essential biological role in pan-cancer. The different gene effects of SLC7A1, SLC7A2, and SLC7A3 in pan-cancer highlight their distinct molecular functions in tumors, emphasizing the significance of further exploration.

As one of the three members of the CAT family, SLC7A1 plays a major role in cellular arginine uptake [7, 17]. SLC7A1 has been reported to promote tumor cell proliferation by regulating cellular uptake of arginine [7, 10, 18]. Feature plot based on the tSNE algorithm showed that SLC7A1 was highly expressed in meningioma cells of clusters 4 and 9 (Fig. 1C, D). To explore the biological processes associated with SLC7A1 expression in meningioma cells, clusters 4 and 9 were classified as high-SLC7A1 clusters, while the rest were classified as low-SLC7A1 clusters. Next, we performed GSVA to calculate the pathway activity score of each meningioma cell based on the 50 cancer hallmark gene sets. A comparison of high-SLC7A1 versus low-SLC7A1 clusters revealed MYC targets, G2M checkpoint, and E2F targets as the top enriched hallmark pathways (Fig. 1F–I), which were in high accordance with the GSEA at the bulk level (Fig. 1J, Supplementary Fig. S3).

### High SLC7A1 expression is related to malignant phenotypes and poor prognosis of meningioma

**Fig. 2:**
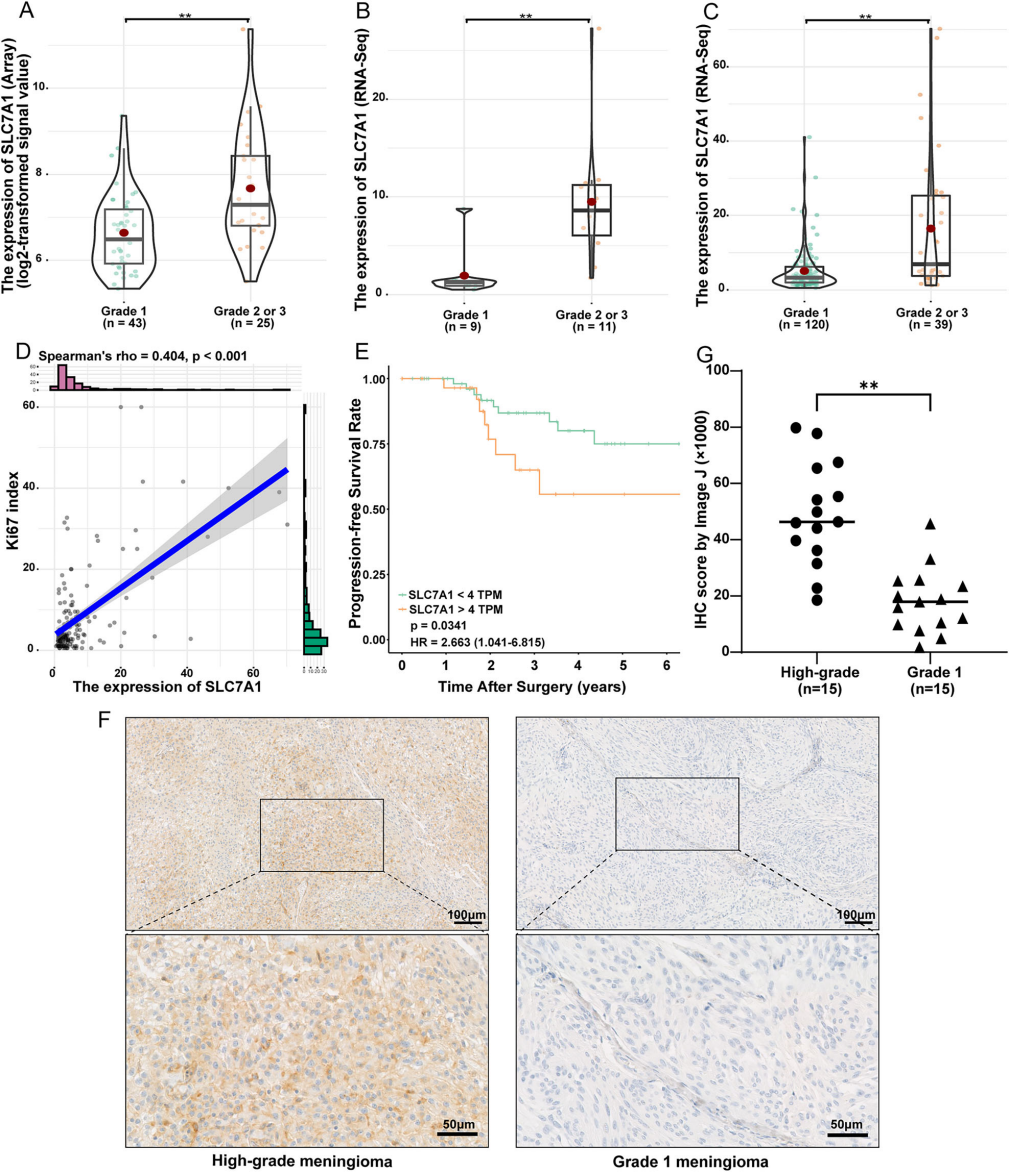
SLC7A1 expression is significantly upregulated in high-grade meningioma. The expression difference of SLC7A1 between grade 1 meningiomas and high-grade meningiomas in the GSE16581 gene array dataset (**A**), our RNA sequencing dataset (**B**), and the GSE136661 RNA sequencing dataset (**C**). **D** Correlation analysis between SLC7A1 expression and Ki67 index in the GSE136661 dataset. **E** Kaplan–Meier survival analysis based on the log-rank test in the GSE136661 dataset. **F** Representative IHC pictures of SLC7A1 expression in high-grade meningiomas and grade 1 meningiomas. **G** The semi-quantitative scoring results of SLC7A1 IHC staining in high-grade meningiomas and grade 1 meningiomas. **P < 0.01.

By analyzing the bulk transcriptomics data of three meningioma datasets, we found that SLC7A1 expression was significantly upregulated in high-grade meningioma (Fig. 2A–C). In addition, the expression level of SLC7A1 was closely associated with tumor proliferation index and patient prognosis. Spearman’s correlation analysis revealed that SLC7A1 expression was positively correlated with ki67 index in meningioma (*r* = 0.404, *P* < 0.001) (Fig. 2D). Kaplan-Meier analysis showed that meningioma patients with SLC7A1 expression >4 TPM experienced a worse prognosis than those with SLC7A1 expression <4 TPM (HR = 2.663, *P* = 0.0341) (Fig. 2E). Furthermore, the differential expression of SLC7A1 between high-grade meningioma and grade 1 meningioma has also been validated at the protein level by IHC (Fig. 2F, G).

Moreover, we performed virtual knockdown analysis using the RNA sequencing data from the GSE136661 dataset to infer the functional role of SLC7A1 in meningioma at the bulk level. GSEA between the high- and low-SLC7A1 groups revealed significant enrichment of cancer hallmark pathways that are closely associated with cell proliferation, such as the G2M checkpoint, E2F targets, and MYC targets (Supplementary Fig. S3, Fig. 1J), which were in high accordance with the GSVA at the single-cell level (Fig. 1F–I). These pathways exhibited a substantial increase in NES, indicating a strong correlation between SLC7A1 expression and tumor proliferation. All these results confirmed that high SLC7A1 expression contributed to malignant phenotypes of meningioma.

### SLC7A1 is a potential antitumor target of meningioma

**Fig. 3:**
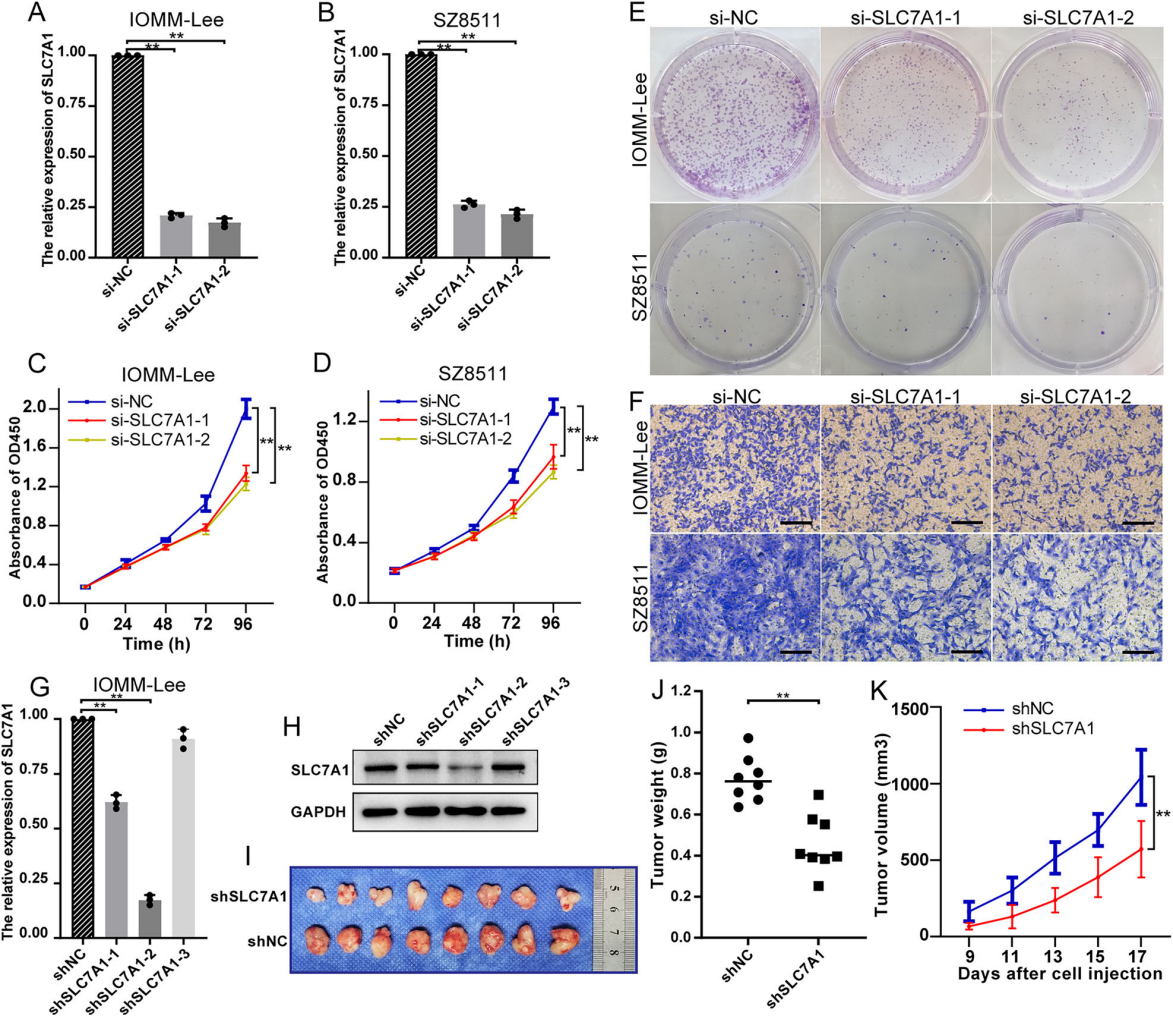
SLC7A1 is a potential antitumor target of meningioma. **A**, **B** Knockdown efficiency of si-SLC7A1 in IOMM-Lee and SZ8511 verified by RT-qPCR. **C**, **D** Effect of SLC7A1 knockdown on the cell proliferation of IOMM-Lee and SZ8511. **E** Effect of SLC7A1 knockdown on the clone formation of IOMM-Lee and SZ8511. **F** Effect of SLC7A1 knockdown on the invasion of IOMM-Lee and SZ8511. **G** Knockdown efficiency of shSLC7A1 in IOMM-Lee verified by RT-qPCR. **H** Knockdown efficiency of shSLC7A1 in IOMM-Lee verified by Western blot. **I** Visual pictures showing representative tumors of each group in vivo (*n* = 8). shSLC7A1-2 was selected for use in animal experiments. **J** Tumor weight of each group. **K** Tumor growth curve displaying the dynamic change of tumor volume of each group. **P < 0.01.

Considering the vital role of SLC7A1 in meningioma, targeting SLC7A1 may be a valuable strategy to treat meningioma. To assess the effect of SLC7A1 on meningioma cells, we knocked down the expression of SLC7A1 with siRNA in vitro. Knockdown efficiency was verified by RT-qPCR (Fig. 3A, B). CCK-8 and clone formation assays showed that the proliferation of meningioma cells was significantly inhibited by SLC7A1 knockdown (Fig. 3C–E). Transwell assay showed that SLC7A1 knockdown also suppressed the invasion of meningioma cells (Fig. 3F). Additionally, in vivo experiments were conducted to validate the function of SLC7A1. Among the three shRNAs tested, shSLC7A1-2 exhibited the highest knockdown efficiency and was therefore utilized in subsequent experiments (Fig. 3G, H). The result showed that knockdown of SLC7A1 significantly inhibited meningioma growth in vivo, further confirming the essential role of SLC7A1 in meningioma (Fig. 3I–K).

### SLC7A1-FOXM1/E2F4 regulatory axis may contribute to the malignant progression of meningioma

**Fig. 4:**
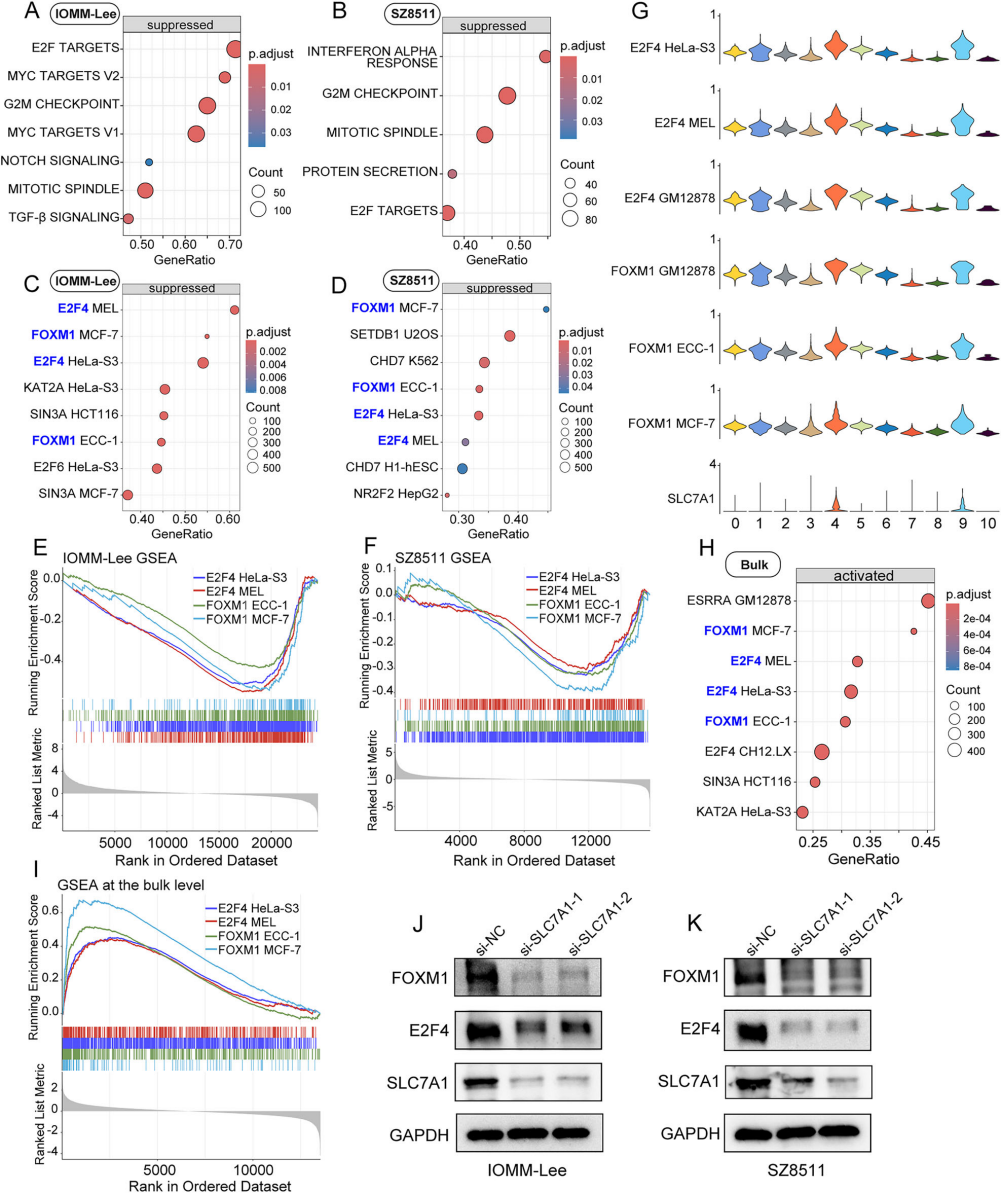
SLC7A1 may exert oncogenic functions in meningioma by regulating transcription factors FOXM1 and E2F4. **A**, **B** GSEA based on the cancer hallmark gene sets to assess the effect of SLC7A1 knockdown on IOMM-Lee and SZ8511. **C**, **D** GSEA based on the transcription factor gene sets to evaluate the impact of SLC7A1 knockdown on transcription factor activity in IOMM-Lee and SZ8511. **E**, **F** GSEA revealing a decreased transcriptional activity of FOXM1 and E2F4 in SLC7A1-knockdown IOMM-Lee and SZ8511. **G** GSVA at the single-cell level revealing the transcriptional activity scores of FOXM1 and E2F4 in different clusters of meningioma cells. **H**, **I** GSEA at the bulk level also revealing an increased transcriptional activity of FOXM1 and E2F4 in the high-SLC7A1 group of the GSE136661 dataset. **J**, **K** Western blot displaying the impact of SLC7A1 knockdown on the protein level of FOXM1 and E2F4 in IOMM-Lee and SZ8511.

To gain deeper insights into the molecular functions of SLC7A1 in meningioma, we conducted mRNA sequencing on meningioma cells IOMM-Lee and SZ8511 after knocking down the expression of SLC7A1. GSEA based on the cancer hallmark gene sets revealed significant inhibition of E2F targets, MYC targets, G2M checkpoint, Notch signaling, and mitotic spindle pathways in SLC7A1-knockdown IOMM-Lee cells (Fig. 4A). Similarly, SLC7A1 knockdown in SZ8511 cells significantly suppressed the G2M checkpoint, mitotic spindle, and E2F targets (Fig. 4B). These enriched pathways are closely associated with the malignant phenotype of tumor cells.

Furthermore, transcription factor activity analysis was performed. The changes in gene expression at the transcriptional level are largely dependent on the functional activity of upstream transcription factors. Transcriptomic data obtained through transcriptome sequencing can be utilized to elucidate the landscape activities of transcription factors. To uncover the transcription factors regulated by SLC7A1 in meningioma cells, we conducted transcription factor activity scoring analysis, referencing the previously reported methods [19, 20]. GSEA based on the transcription factor gene sets showed a significant decrease in the transcriptional activity of FOXM1 and E2F4 in both SLC7A1-knockdown IOMM-Lee and SZ8511 cells (Fig. 4C–F). FOXM1 has been identified as a key target in meningiomas [21]; however, the role of E2F4 in meningioma has not been previously reported. To assess the effect of E2F4 on meningioma cells, CCK‑8 assays were performed after siRNA‑mediated knockdown of E2F4. The results showed that E2F4 knockdown significantly inhibited the proliferation of meningioma cells, suggesting that E2F4 may contribute to the malignant progression of meningioma (Supplementary Fig. S4).

Interestingly, GSVA analysis at the single-cell level also revealed a significant increase in the transcriptional activity scores of FOXM1 and E2F4 in clusters 4 and 9 of meningioma cells with high SLC7A1 expression (Fig. 4G). In addition, the bulk-level GSEA analysis also demonstrated a significant upregulation of transcriptional activity for FOXM1 and E2F4 in meningioma samples from the high-SLC7A1 group compared to the low-SLC7A1 group (Fig. 4H, I). Furthermore, we validated these findings at the protein level in meningioma cells. The results revealed a significant reduction of FOXM1 and E2F4 upon SLC7A1 knockdown in meningioma cells (Fig. 4J, K). All these findings suggest that the SLC7A1-FOXM1/E2F4 regulatory axis may contribute to the malignant progression of meningioma.

In addition, GSEA analysis revealed that knockdown of SLC7A1 in IOMM-Lee and SZ8511 cells led to an increase in the transcriptional activity of the transcription factor REST (Supplementary Tables S3, S4, Supplementary Fig. S5A, B), which is consistent with the results of transcription factor activity scoring at the single-cell level (Supplementary Fig. S5C). However, further validation is required to determine whether REST functions as a tumor suppressor gene in meningioma.

### AZ628 is a potential small molecule drug that targets high-SLC7A1 meningiomas

**Fig. 5:**
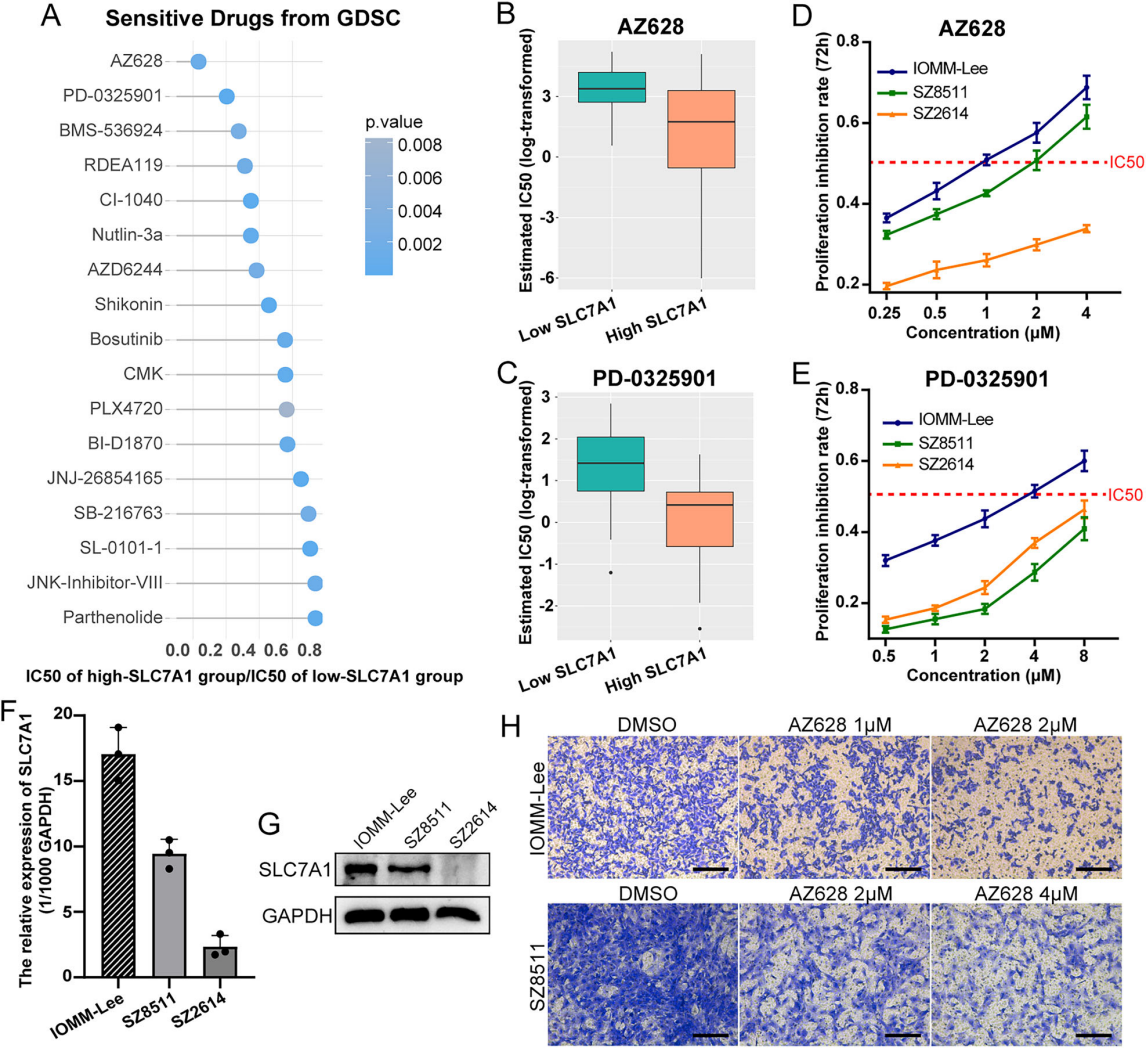
AZ628 is a potential small molecule drug that targets high-SLC7A1 meningiomas. **A** Potential drugs targeting high-SLC7A1 meningiomas predicted by GDSC. **B**, **C** The top two drugs predicted with the highest sensitivity to high-SLC7A1 meningiomas. **D**, **E** The proliferation inhibition rate of AZ628 and PD-0325901 on meningioma cells at 72 h. **F**, **G** The mRNA and protein expression levels of SLC7A1 in meningioma cells IOMM-Lee, SZ8511, and SZ2614. **H** Effect of AZ628 treatment on the invasion of IOMM-Lee and SZ8511.

Based on the drug sensitivity data from the GDSC database, we predicted the potential drugs targeting SLC7A1 expression in meningioma. The sensitivity index of the predicted drugs was determined by the ratio of IC50 of the high-SLC7A1 group to that of the low-SLC7A1 group. We found 17 drugs with lower IC50 in the high-SLC7A1 group (Fig. 5A), indicating that they might have better antitumor effects on meningiomas with high expression of SLC7A1. AZ628 and PD-0325901 were the top two drugs with the highest sensitivity to high-SLC7A1 meningiomas (Fig. 5A–C). Next, we conducted the CCK-8 assay to evaluate the antitumor effect of AZ628 and PD-0325901 on three meningioma cells in vitro. CCK-8 assay verified that AZ628 and PD-0325901 could inhibit the proliferation of meningioma cells (Fig. 5D, E). It should be noted that AZ628 exhibited a significantly better anti-proliferative effect compared to PD-0325901, underscoring its potential candidacy as a more promising compound. The effects of AZ628 on normal dura mater cells were also assessed. CCK-8 assays demonstrated a significantly weaker proliferation-inhibitory effect of AZ628 on dura mater cells compared to meningioma cells (Supplementary Fig. S6).

Additionally, the inhibitory effect of AZ628 on proliferation varied across different meningioma cells. AZ628 exhibited obviously stronger inhibition of cell proliferation in IOMM-Lee and SZ8511 compared to SZ2614 (Fig. 5D). Expression analysis revealed higher SLC7A1 expression in IOMM-Lee and SZ8511 compared to SZ2614 (Fig. 5F, G), further indicating the targeted inhibitory effect of AZ628 on meningiomas with high SLC7A1 expression. Moreover, we evaluated the effect of AZ628 on the invasion of meningioma cells. The working concentration of AZ628 was determined based on the IC50 value specific to each cell. Transwell assay demonstrated that AZ628 also significantly inhibited the invasion of meningioma cells (Fig. 5H).

**Fig. 6:**
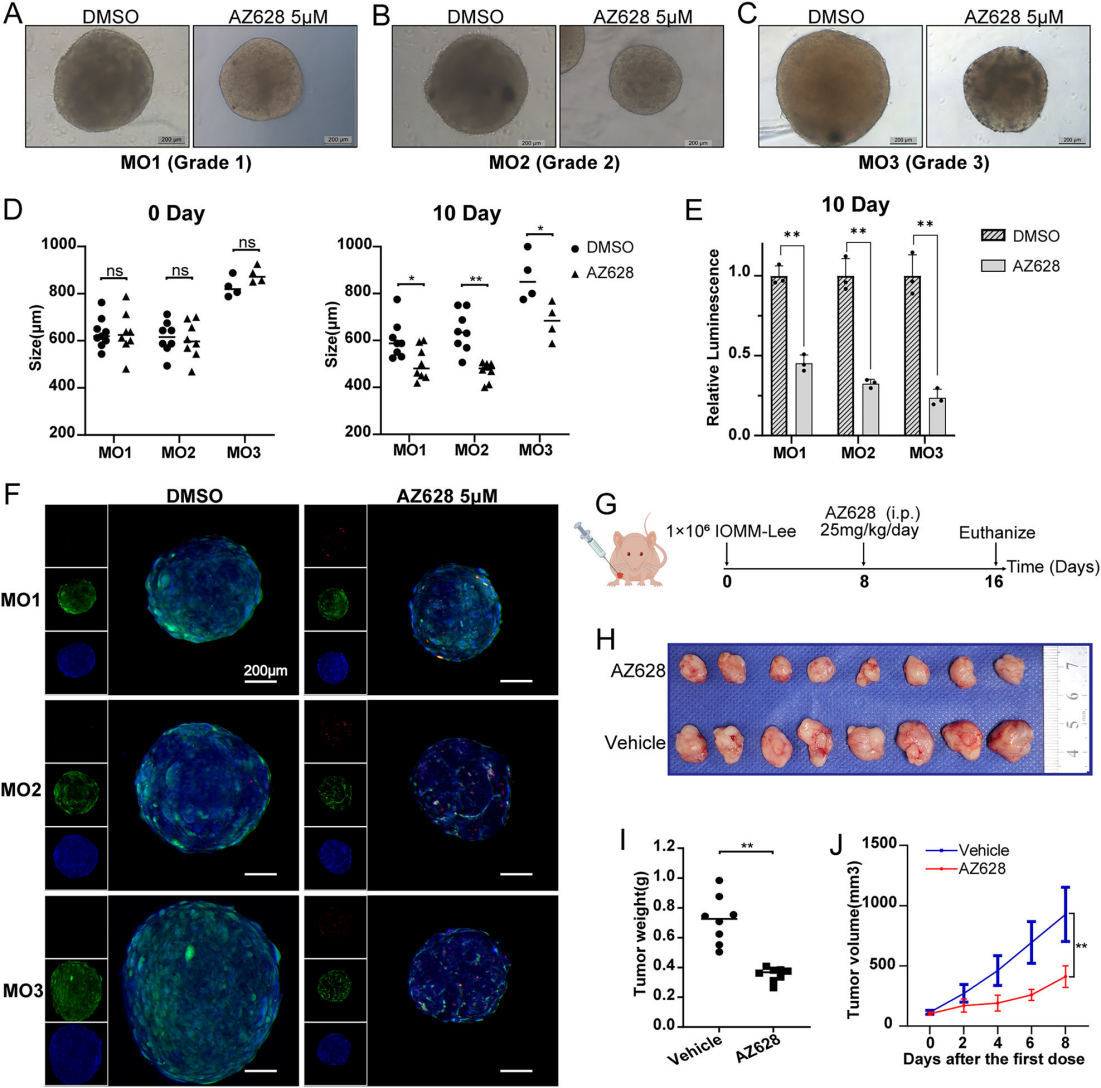
AZ628 inhibits the growth of meningioma in both organoid and animal experiments. **A**–**C** Bright-field images of meningioma organoids of different grades after treatment with DMSO or AZ628 for 10 days. Scale bars indicate 200 μm. MO meningioma organoid. **D** Effect of AZ628 treatment on the growth of meningioma organoids of different grades after treatment with DMSO or AZ628 for 10 days. The size of the organoid is determined by the average of the length and width. **E** CellTiter-Glo 3D Cell Viability Assay demonstrating AZ628’s proliferation-inhibitory effect on meningioma organoids. **F** Calcein‑AM/PI staining of meningioma organoids after treatment with AZ628. Representative confocal images show increased necrotic cells (PI-positive, red fluorescence) in AZ628‑treated organoids compared to vehicle‑treated controls. Green fluorescence denotes live cells (Calcein‑AM‑positive), and blue fluorescence marks nuclei (DAPI). **G** Schematic diagram of the administration procedure of AZ628 in tumor-bearing mice. **H** Visual pictures showing representative tumors of each group (*n* = 8). **I** Tumor weight of each group. **J** Tumor growth curve displaying the dynamic change of tumor volume of each group. *p < 0.05, **P < 0.01, ns not significant.

In addition, meningioma organoids were also used to evaluate the antitumor effect of AZ628. We selected one case each of WHO grade 1, grade 2, and grade 3 meningioma patients for organoid culture and drug testing. The clinical information of the meningioma patients used for organoid generation is listed in Supplementary Table S5. The results demonstrated that treatment with 5 μM AZ628 significantly inhibited the growth of meningioma organoids (Fig. 6A–D). The CellTiter-Glo 3D Cell Viability Assay further confirmed the proliferation-inhibitory effect of AZ628 on meningioma organoids (Fig. 6E). Calcein-AM/PI staining was also performed to assess the cytotoxic effect of AZ628 on meningioma organoids. The results demonstrated that AZ628 significantly increased necrosis in meningioma organoids (Fig. 6F).

Furthermore, in vivo experiment confirmed that AZ628 treatment effectively suppressed meningioma growth (Fig. 6G–J). Additionally, no significant differences were observed in the pathological examination of the heart, lung, liver, spleen, kidney, and colon between the AZ628 treatment group and the control group (Supplementary Fig. S7).

### AZ628 may exert anti-meningioma effect by inhibiting FOXM1 and E2F4

**Fig. 7:**
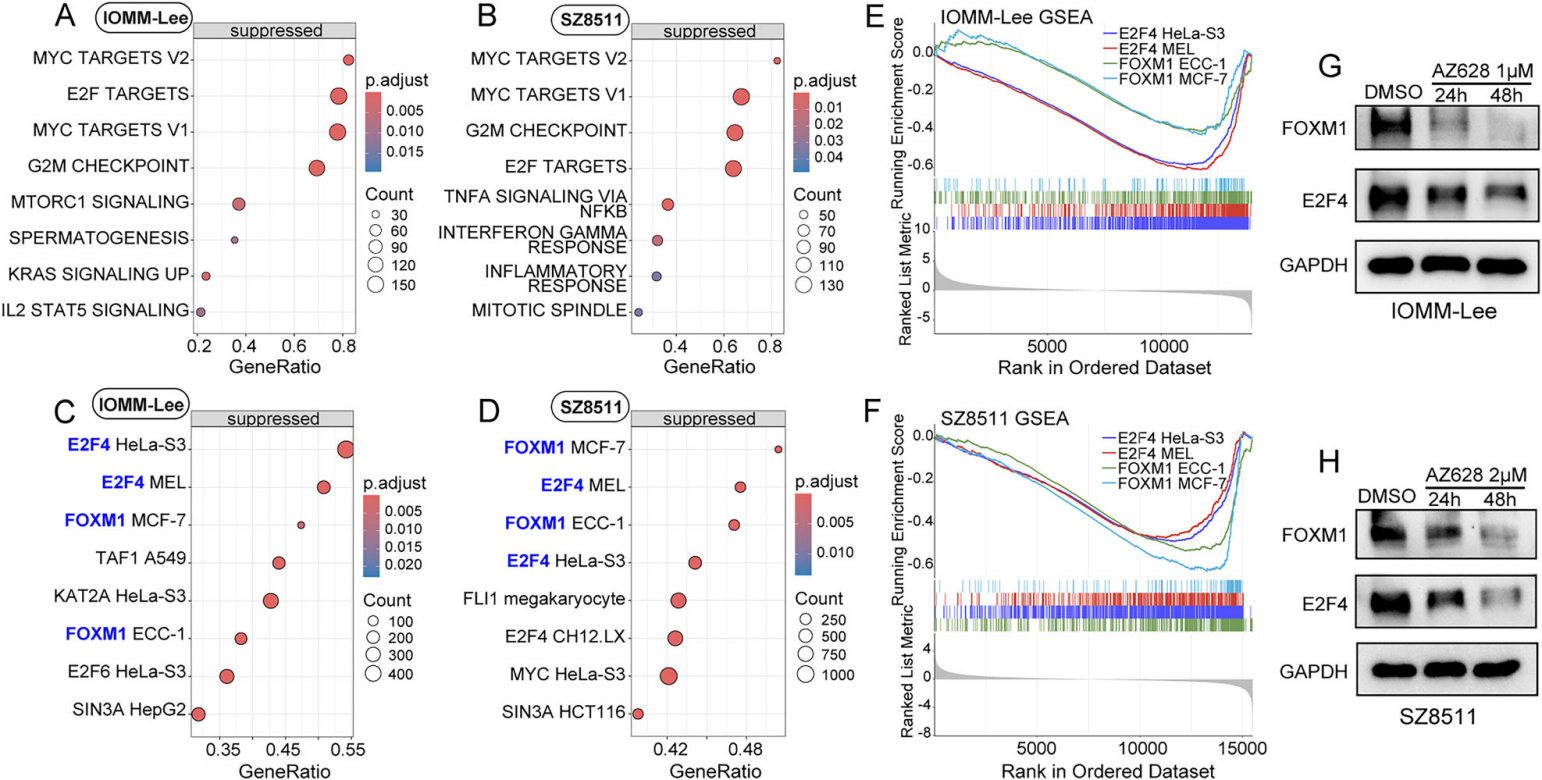
AZ628 may exert anti-meningioma effect by inhibiting FOXM1 and E2F4. **A**, **B** GSEA based on the cancer hallmark gene sets to assess the effect of AZ628 treatment on IOMM-Lee and SZ8511. **C**, **D** GSEA based on the transcription factor gene sets to evaluate the impact of AZ628 treatment on transcription factor activity in IOMM-Lee and SZ8511. **E**, **F** GSEA revealing a decreased transcriptional activity of FOXM1 and E2F4 in AZ628-treated IOMM-Lee and SZ8511. **G**, **H** Western blot displaying the impact of AZ628 treatment on the protein level of FOXM1 and E2F4 in IOMM-Lee and SZ8511.

To further explore the signaling pathways intervened by AZ628, we performed mRNA sequencing on AZ628-treated meningioma cells IOMM-Lee and SZ8511. GSEA based on the cancer hallmark gene sets revealed significant inhibition of MYC targets, E2F targets, G2M checkpoint, and mTORC1 signaling pathways in AZ628-treated IOMM-Lee cells (Fig. 7A). Similarly, AZ628 treatment in SZ8511 cells significantly inhibited MYC targets, G2M checkpoint, E2F targets, and TNFα-NFKB signaling pathways (Fig. 7B).

**Fig. 8:**
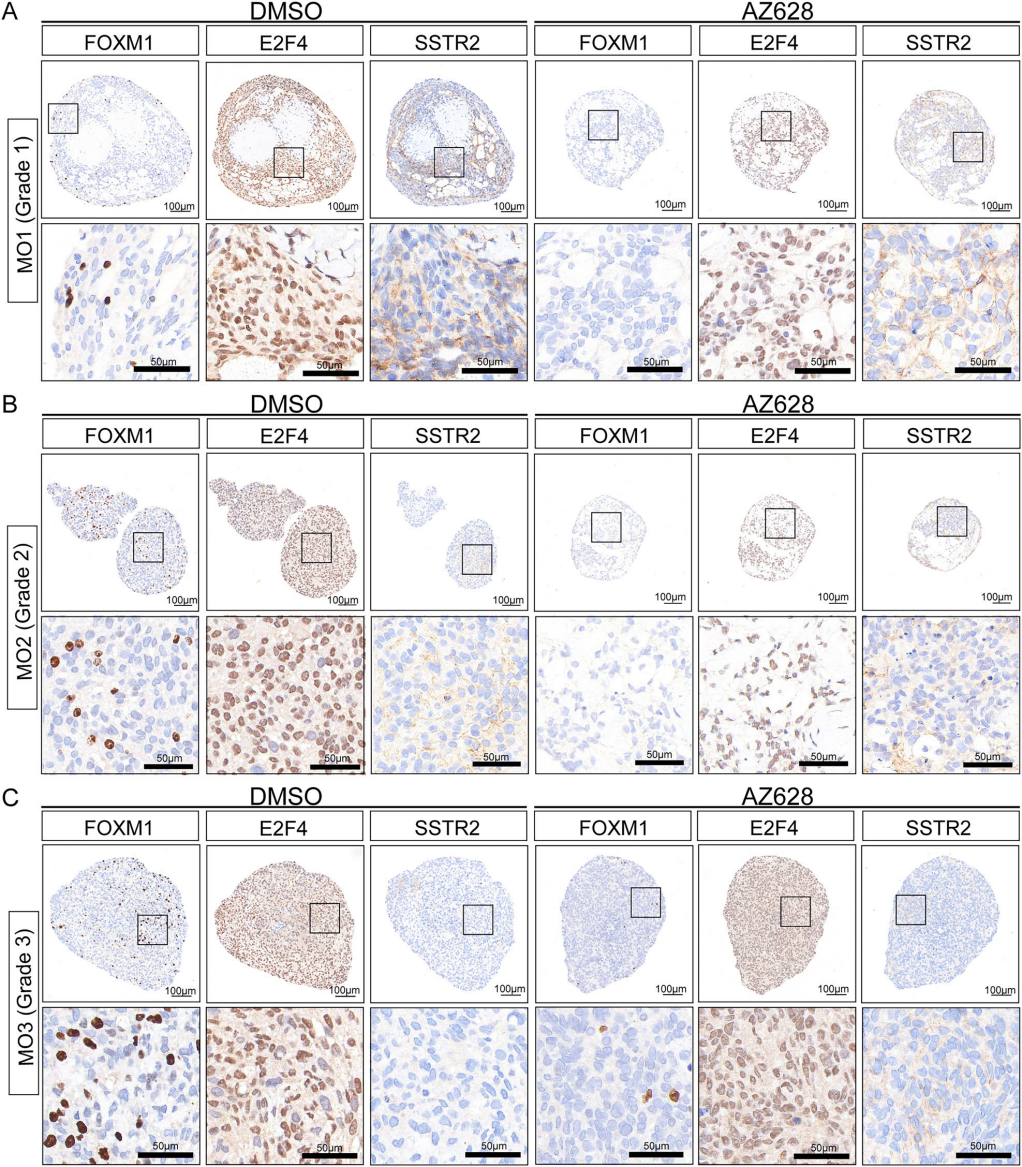
AZ628 treatment decreases the expression of FOXM1 and E2F4 in meningioma organoids. Representative photographs of immunohistochemical staining for FOXM1, E2F4, and SSTR2 in organoids derived from WHO grade 1 (**A**), grade 2 (**B**), or grade 3 meningioma (**C**), after treatment with DMSO or AZ628 (5 μM) for 10 days. Scale bars indicate 100 μm (upper panels) and 50 μm (lower panels).

Additionally, we performed GSEA based on the transcription factor gene sets. The results showed a significant decrease in the transcriptional activity of FOXM1 and E2F4 in AZ628-treated IOMM-Lee and SZ8511 cells (Fig. 7C–F), which is similar to the effect observed with SLC7A1 knockdown (Fig. 4C–F). Western blot confirmed a progressive downregulation of FOXM1 and E2F4 with increasing duration of AZ628 treatment in meningioma cells (Fig. 7G, H). Furthermore, it can also be observed in the meningioma organoids that treatment with AZ628 leads to the downregulation of FOXM1 and E2F4 (Fig. 8A–C). In addition, we noted a downregulation in the transcriptional levels of SLC7A1 in IOMM-Lee and SZ8511 cells treated with AZ628 (Supplementary Fig. S8A, B). After 48 h of AZ628 treatment, the protein level of SLC7A1 was also decreased (Supplementary Fig. S8C), suggesting the targeted effect of AZ628 against SLC7A1.

## Discussion

High-grade meningioma remains a therapeutic challenge to neurosurgeons and oncologists. The absence of first-line guideline drugs highlights an urgent need to explore the essential players that contribute to the progression and malignancy of meningioma. Here, we identified the potential presence of urea cycle disorder and arginine auxotrophy in meningioma. As an arginine transporter, SLC7A1 showed significant correlation with malignant phenotypes in meningioma both at the single-cell level and bulk level. SLC7A1 was highly expressed in high-grade meningioma and associated with poor prognosis of patients. Moreover, we demonstrated that SLC7A1 regulates multiple signaling pathways involved in tumor proliferation, including E2F targets, G2M checkpoint, and MYC targets, in meningioma. Additionally, SLC7A1 may exert oncogenic functions in meningioma by regulating transcription factors FOXM1 and E2F4. These findings provide SLC7A1 as a potential therapeutic target for high-grade meningioma.

SLC7A1 is a membrane transporter responsible for the uptake of arginine [16]. Arginine is a semiessential amino acid that plays a crucial role in cellular metabolism and homeostasis [8]. Increased intracellular arginine level is associated with chemoresistance and tumor progression [7, 10, 11, 22]. Arginine deprivation can lead to a decrease in mTORC activity [7]. Although several studies have demonstrated the oncogenic role of SLC7A1 in various cancers, the downstream pathways and molecular targets regulated by SLC7A1 remain largely unclear, and its function in meningioma is yet to be elucidated. In this study, we analyzed the signaling pathways that may be regulated by SLC7A1 at both the single-cell and bulk levels. We found a close correlation between SLC7A1 expression and cell proliferation-related pathways, including E2F targets, G2M checkpoint, and MYC targets. Consistently, similar results were obtained from RNA sequencing analysis after knocking down SLC7A1 in meningioma cells, further suggesting the potential oncogenic role of SLC7A1 in meningioma through the regulation of E2F targets, G2M checkpoint, and MYC targets pathways.

In addition, it is worth mentioning that, apart from the crucial role in tumor cell, arginine also plays a vital role in the functional activity of T cell [14]. Insufficient supply of arginine leads to decreased expression of CD3 and T cell receptor, reduced proliferation, and impaired immune activity of T cell [15]. Previous studies have reported that tumor cells with high expression of transporter proteins can compete with immune cells for amino acids, thereby impairing the function of immune cells [23–25]. Tumor-associated arginine deprivation may impair T cell-mediated antitumor immune responses and promote tumor immune escape. For instance, neuroblastoma creates an arginine-depleted microenvironment by increasing arginase activity, leading to suppression of T cell proliferation [26]. Ovarian cancer cells utilize extracellular vesicles to deliver arginase ARG1 to immune cells, which inhibits the proliferation of CD4+ and CD8+ T cells by affecting their arginine metabolism, thereby suppressing antitumor immune responses [27]. As one of the three members of the CAT family, SLC7A1 plays a major role in cellular arginine uptake [7, 17]. Overexpression of SLC7A1 in meningioma cells may also create an arginine-depleted microenvironment by enhancing arginine uptake into tumor cells. However, this hypothesis still requires further validation.

In vitro and in vivo experiments demonstrated that knockdown of SLC7A1 significantly inhibited the proliferation, invasion, and xenograft tumor growth of meningioma cells. RNA sequencing analysis revealed a significant decrease in the transcriptional activity of FOXM1 and E2F4 in IOMM-Lee and SZ8511 meningioma cells following SLC7A1 knockdown. Moreover, GSVA at the single-cell level and GSEA at the bulk level consistently showed a close association between SLC7A1 expression and the transcriptional activity of FOXM1 and E2F4. Furthermore, subsequent experimental validation confirmed the downregulation of FOXM1 and E2F4 at the protein level following SLC7A1 knockdown. Excitingly, accumulating evidences have already established FOXM1 as a critical transcription factor involved in the malignant progression of meningioma. Vasudevan et al. reported that FOXM1 is a key transcription factor for meningioma proliferation and a marker of poor prognosis [28]. The activation of the FOXM1/Wnt signaling axis was observed in aggressive meningiomas [28]. Kim et al. demonstrated the crucial role of FOXM1 in meningioma growth through cell and animal experiments [29]. Yamazaki et al. confirmed the vital role of FOXM1 in meningioma using organoid experiment [30]. Furthermore, an integrative epigenomic analysis based on DNA methylation in meningioma conducted by Choudhury et al. highlighted the critical function of FOXM1 in the malignant progression of meningioma [21]. Although there is no specific research on E2F4 in meningioma, it is widely recognized as an oncogenic transcription factor in various tumors [31–33]. We also verified by CCK-8 assays that knockdown of E2F4 significantly suppresses the proliferation of meningioma cells, indicating that E2F4 may contribute to the malignant progression of meningioma. These findings suggest that the SLC7A1-FOXM1/E2F4 regulatory axis may contribute to the malignant progression of meningioma. It is worth noting that MYC has been demonstrated as a downstream target of both FOXM1 and E2F4 [34, 35]. As key regulators of the cell cycle, FOXM1 and E2F4 also play crucial roles in the G2M and E2F targets pathways [36, 37]. Therefore, SLC7A1 may facilitate the malignant progression of meningioma through the regulation of E2F targets, G2M checkpoint, and MYC targets pathways via the SLC7A1-FOXM1/E2F4 axis. However, further research is needed to elucidate the molecular mechanisms underlying the regulation of transcription factors FOXM1 and E2F4 by SLC7A1 in meningioma.

Furthermore, this study provides a novel therapeutic strategy for high-grade meningioma. We predicted the potential drugs targeting SLC7A1 expression in meningioma. GDSC analysis combined with experimental validation showed that AZ628 was a potential antitumor drug for high-SLC7A1 meningioma. AZ628 is a type II selective and potent pan-Raf kinase inhibitor under clinical pipeline by AstraZeneca [38]. The signaling pathways and molecular targets regulated by AZ628 in meningioma were further explored through RNA sequencing. The pathways inhibited by AZ628 treatment mainly included MYC targets, E2F targets, and G2M checkpoint, which exhibited similarities to the effect of SLC7A1 knockdown. Additionally, AZ628 treatment significantly suppressed the transcriptional activity and protein level of FOXM1 and E2F4. These findings suggest that AZ628 treatment can partially mimic the effect of SLC7A1 knockdown in meningioma. Furthermore, AZ628 treatment also inhibited the expression of SLC7A1 in meningioma cells, indicating that AZ628 is a potential anti-meningioma drug that targets SLC7A1.

In conclusion, targeting SLC7A1 emerges as a promising strategy for the treatment of high-grade meningioma. SLC7A1 may facilitate the malignant progression of meningioma through the regulation of E2F targets, G2M checkpoint, and MYC targets pathways via the SLC7A1-FOXM1/E2F4 axis. Additionally, the small molecule drug AZ628 holds potential as a therapeutic agent for high-grade meningioma.

## Materials and methods

### Data processing of single-cell RNA sequencing (scRNA-seq)

The scRNA-seq data of meningioma samples were downloaded from the GEO database (GEO accession: GSE183655). A total of 49254 cells from six patients were included in the analysis. R package “Seurat” was applied to process the scRNA-seq data. We excluded poor-quality cells based on the following cell-filtering parameters: 500 < nFeature_RNA < 6000, percent.mt < 20%. After filtering, 32714 cells remained. Subsequently, the top 2000 highly variable genes were selected for further analysis. Cell clusters were visualized by T-distributed Stochastic Neighbor Embedding (tSNE) with RunTSNE function (dims = 1:20).

### Gene set variation analysis (GSVA)

GSVA was used to calculate the activity scores of hallmark pathways and transcription factors at the single-cell level. The cancer hallmark gene sets (h.all.v7.5.1.symbols, containing 50 cancer hallmarks) were downloaded from the MSigDB database [39]. The transcription factor gene sets (ENCODE_TF_ChIP-seq_2015, containing 816 ChIP-seq results of various transcription factors) were downloaded from the Enrichr database [40]. We calculated the activity score of each cell based on the cancer hallmark gene sets and transcription factor gene sets using R package “GSVA”. Subsequently, we analyzed the differences in activity scores between different cell clusters using R package “limma”.

### Datasets of bulk transcriptomics

To compare the expression of SLC7A1 in different grades of meningioma, we downloaded transcriptomic data from two datasets: a gene array dataset (GEO accession: GSE16581) and an RNA sequencing dataset (GEO accession: GSE136661), both sourced from the GEO database. The corresponding clinical information for these datasets was obtained from previously published studies [41–43]. Additionally, an RNA sequencing dataset containing 20 meningioma samples from our previous study was also included in the analysis [44].

### Bulk RNA sequencing analysis

The GSE136661 dataset is the largest collection of RNA sequencing data for meningioma available in the GEO database, and it was utilized to analyze the molecular function of SLC7A1 in meningioma at the bulk level. The count data was transformed into TPM (Transcripts Per Million) format for subsequent analysis. Patients with SLC7A1 expression higher than 10 TPM were divided into the high-SLC7A1 group, while those with SLC7A1 expression lower than 2 TPM were divided into the low-SLC7A1 group. The remaining patients were divided into the medium-SLC7A1 group. Differential expression analysis between the high- and low-SLC7A1 groups was performed to simulate the virtual knockdown of SLC7A1 and infer the molecular function of SLC7A1 at the bulk level. Then gene set enrichment analysis (GSEA) based on the cancer hallmark gene sets (h.all.v7.5.1.symbols) and transcription factor gene sets (ENCODE_TF_ChIP-seq_2015) was conducted using R packages “fgsea” and “clusterProfiler”. Normalized enrichment score (NES) was applied to evaluate the gene set enrichment.

### Chemotherapeutic response prediction

To explore the potential drugs targeting SLC7A1 expression in meningioma, we predicted the chemotherapeutic response of meningioma samples in the high- and low-SLC7A1 groups of the GSE136661 dataset. The prediction process was conducted based on the Genomics of Drug Sensitivity in Cancer (GDSC) database using R package “pRRophetic”. Log-normalized 50% inhibitory concentration (IC50) values generated by GDSC were used for comparison. AZ628 (HY-11004, MCE, NJ, USA) and PD0325901 (HY-10254, MCE) were selected for further experimental validation.

### Immunohistochemistry (IHC)

All paraffin-embedded meningioma samples were cut into 5-μm thick slices. Antigen retrieval was performed according to the instruction manual of antibodies. After inactivating endogenous peroxidase, the slides were incubated with protein blocking buffer (ab64226, Abcam, UK) for 30 min at room temperature. Then, the slides were incubated overnight with anti-SLC7A1 antibody (PA5-90039, Invitrogen, CA, USA) at 4 °C. The secondary antibody (PR30009, Proteintech, China) was incubated for 30 min at room temperature. The semi-quantitative analysis of immunohistochemical staining was performed using the Image J Fiji software, following a previously reported protocol [45].

### Western blot

Total proteins of meningioma cells were extracted using RIPA lysis buffer at 4 °C and then quantified using a BCA Protein Assay Kit (Solarbio, Beijing, China). Next, equal amounts of proteins were loaded and subjected to electrophoresis. Following blocking, the membrane was incubated with primary antibody overnight at 4 °C. Subsequently, secondary antibody was incubated for 60 min at room temperature. The protein bands were detected using the ECL Western Blotting Substrate (Solarbio). Antibodies against FOXM1 (ab207298, Abcam), E2F4 (ab150360, Abcam), SLC7A1 (PA5-90039, Invitrogen), and GAPDH (60004-1-Ig, Proteintech) were used according to the manufacturer’s instructions. Full length uncropped original western blots are uploaded in Supplementary Files.

### Culture of meningioma cells and organoids

Human malignant meningioma cell line IOMM-Lee was cultured in DMEM (Gibco, NY, USA) supplemented with 10% fetal bovine serum (FBS; Gibco, NY, USA). In addition, two primary meningioma cells SZ8511 and SZ2614, which were established from two different patients with high-grade meningioma, were also used for the cellular experiment. Meningioma cell markers Vimentin and EMA were used for cell verification [46]. SZ8511 and SZ2614 have been immortalized by expression of SV40 large T antigen with reference to previously reported method [46, 47]. The clinical information of meningioma patients used for primary meningioma cell culture is provided in Supplementary Table S1. The immortalized meningioma cells were cultured in IMDM (Gibco): RPMI-1640 (Gibco) (4:1) supplemented with 10% FBS (Gibco) and 1× Pen Strep (Gibco). The staining results of molecular markers Vimentin and EMA in immortalized meningioma cells are presented in Supplementary Fig. S1. All cell lines have undergone STR profiling to prevent cross-contamination. No mycoplasma was detected in any of the cell lines. Meningioma organoids were established according to a previously reported method [30]. The clinical information of the meningioma patients used for organoid generation is provided in Supplementary Table S4. This study was approved by the Institutional Review Boards of Beijing Tiantan Hospital and informed consent was obtained from all patients.

### Cell transfection

For transient SLC7A1 silencing, meningioma cells were transfected with siRNA using lipo3000 (Invitrogen). si-SLC7A1-1, si-SLC7A1-2, and negative control siRNA (si-NC) were purchased from RiboBio Company (Guangzhou, China). For stable SLC7A1 knockdown, meningioma cells were transfected with GV493 lentiviruses containing short hairpin RNA (shRNA) targeting SLC7A1. SLC7A1 knockdown and negative control lentiviruses were purchased from GeneChem Company (Shanghai, China). The sequences of siRNAs and shRNAs are presented in Supplementary Table S2.

### Real-time quantitative PCR (RT-qPCR)

RT-qPCR was performed according to previously described protocols [48]. SLC7A1 was normalized to GAPDH as an endogenous control. The primer sequences are also listed in Supplementary Table S2.

### mRNA sequencing and data analysis

The library preparation and mRNA sequencing of meningioma cells IOMM-Lee and SZ8511 were done by Gene Denovo (Guangzhou, China). In brief, 1 μg total RNA was used for cDNA library construction using NEBNext Ultra RNA Library Prep Kit (NEB #7530, New England Biolabs, MA, USA). After end repair, A-base addition, adaptor ligation, and PCR amplification, the sequencing libraries were multiplexed and loaded on an Illumina HiSeq instrument for sequencing using a 2 × 150 paired-end configuration. Differential expression analysis of gene count data was conducted using R package “DESeq2”. Subsequently, GSEA based on the cancer hallmark gene sets and transcription factor gene sets was performed using R package “clusterProfiler”. RNA sequencing data are available in GEO database under accession number GSE244554.

### Cell proliferation assay

Meningioma cells were seeded into 96-well plates at a density of 2 × 10^3^ cells per well. After overnight incubation, siRNA transfection or drug treatment was performed. Cell viability was assessed with Cell Counting Kit-8 (CCK-8, Dojindo, Kumamoto, Japan). For clone formation assay, transfected meningioma cells were seeded into 6-well plates at a density of 1 × 10^3^ cells per well. After 14 days, colonies were stained with 0.1% crystal violet.

### Cell invasion assay

The cell invasion assay was performed using Transwell invasion chambers (Corning) coated with Matrigel (BD, MA, USA). Cells were resuspended in 200 μL serum-free medium at a density of 4 × 10^5^/ml and seeded in the upper chamber. 600 μL culture medium containing 15% FBS was added to the bottom chamber as an attractant. After 24 hours, the invaded meningioma cells were detected using crystal violet staining.

### Organoid viability assays

To evaluate the proliferation-inhibitory effect of AZ628 on meningioma organoids, the CellTiter-Glo® 3D Cell Viability Assay (Promega, WI, USA) was used. According to the manufacturer’s instructions, meningioma organoids were incubated with the assay reagent for 30 min, followed by measurement of luminescence. In addition, Calcein-AM/PI staining was performed to assess the cytotoxic effect of AZ628 on meningioma organoids, using the Beyo3D™ Calcein/PI Cell Viability and Cytotoxicity Assay Kit (Beyotime, Shanghai, China) according to the manufacturer’s instructions.

### Xenograft experiment

Six-week-old female Balb/c nude mice were purchased from SiPeiFu Biotechnology (Beijing, China). For the SLC7A1 silencing experiment, 1 × 10^6^ IOMM-Lee cells transfected with shNC or shSLC7A1 were subcutaneously implanted into the right axilla. After tumor formation, tumor length and width were measured every two days to estimate tumor volume. For the drug treatment experiment, 1 × 10^6^ IOMM-Lee cells were subcutaneously implanted into the right axilla. Once the tumor volume reached ~100 mm^3^, the mice were randomly divided into two groups. The mice were treated with AZ628 (25 mg/kg/day) or an equivalent dose of vehicle by intraperitoneal injection for consecutive five days. When the tumor volume of the control group reached ~1 × 10^3^ mm^3^, all mice were sacrificed via cervical dislocation. No blinding method was used for animal studies. Tumor volume was estimated according to the formula: Volume = length (mm) × width (mm) × width (mm)/2. This study was approved by the Institutional Animal Care and Use Committee of Beijing Neurosurgical Institute.

### Statistics

All statistical analyses were performed using R software version 4.3.1 or GraphPad Prism 9.0. All experiments were repeated three times with at least three replicates. All data were presented as mean ± standard deviation. Differences between two groups were assessed using unpaired two-sided Student’s *t* tests under the assumption of normality; otherwise, the Mann–Whitney test was used. Correlation analyses were performed using Spearman correlation analysis. Two-way ANOVA was utilized to evaluate the intergroup differences in both the in vitro cell proliferation curve and the in vivo tumor growth curve. *P* value (two-sided) <0.05 was considered statistically significant. Sample sizes were determined by referencing the previous studies without prior power analysis.

## Supplementary information

Supplementary Tables and Figures

un-cropped images of the original western blots

## Data Availability

RNA sequencing data in this study are available in GEO database under accession number GSE244554.

## Code availability

The code is available from the corresponding author upon reasonable request.
