# Supplementary material for: Identification of SLC7A1 as a potential therapeutic target for high-grade meningioma
Source: Cell Death Discov. 2025 Nov 3;11:498. doi: 10.1038/s41420-025-02783-4 (PMC12583633; doi:10.1038/s41420-025-02783-4)

**Supplementary Tables**

| **Table S1. Clinical information of the high-grade meningioma patients used for primary meningioma cell culture.**   \| ID \| Sex \| Age  (yrs) \| Location \| WHO  grade \| Histotype \| Ki-67 \| \| --- \| --- \| --- \| --- \| --- \| --- \| --- \| \| SZ2614 \| M \| 63 \| Occipital \| 3 \| Anaplastic \| 30-40% \| \| SZ8511 \| M \| 52 \| Frontal \| 2 \| Atypical \| 5-10% \|   **Table S2. The sequences of siRNAs, shRNAs, and primers.** | |
| --- | --- | --- | --- | --- | --- | --- | --- | --- | --- | --- | --- | --- | --- | --- | --- | --- | --- | --- | --- | --- | --- | --- |
| Name | Sequence |
| si-SLC7A1-1 | 5'-GCATCTTCGTGAACGTCTA-3' |
| si-SLC7A1-2 | 5'-CAGCTTACCTCTACAGCTA-3' |
| shSLC7A1-1 | 5'-CTGGGCTAATTGTGAACATTT-3' |
| shSLC7A1-2 | 5'-GCTGAGGATGGACTGCTATTT-3' |
| shSLC7A1-3 | 5'-CCTACATCATCGGTACTTCAA-3' |
| SLC7A1 forward | 5'-ATCATCGGTACTTCAAGCGTAGC-3' |
| SLC7A1 reverse | 5'-GGCGTTCAGAGTCATGTGTGT-3' |
| GAPDH forward | 5'-ACCCACTCCTCCACCTTTGAC-3' |
| GAPDH reverse | 5'-TGTTGCTGTAGCCAAATTCGTT-3' |

| **Table S3. The activated transcription factors in SLC7A1-knockdown IOMM-Lee.** | | | | |
| --- | --- | --- | --- | --- |
| ID | setSize | enrichmentScore | NES | pvalue |
| REST HCT116 | 211 | 0.376472349 | 1.337616 | 0.019922 |
| EZH2 myotube | 1016 | 0.343712061 | 1.296122 | 0.000303 |
| REST U-87 MG | 256 | 0.348914614 | 1.255835 | 0.049698 |
| EZH2 keratinocyte | 1013 | 0.321239786 | 1.211402 | 0.007404 |
| EZH2 fibroblast of lung | 1212 | 0.31040154 | 1.172763 | 0.016011 |
| EZH2 mammary epithelial cell | 1177 | 0.307037359 | 1.159865 | 0.024012 |

| **Table S4. The activated transcription factors in SLC7A1-knockdown SZ8511.** | | | | |
| --- | --- | --- | --- | --- |
| ID | setSize | enrichmentScore | NES | pvalue |
| SUZ12 NT2-D1 | 326 | 0.6147682 | 2.406651 | 2.91E-23 |
| EZH2 B cell | 1505 | 0.506997959 | 2.134836 | 4.86E-48 |
| REST Panc1 | 439 | 0.463247358 | 1.855265 | 4.92E-10 |
| REST HCT116 | 260 | 0.473119226 | 1.816438 | 4.77E-07 |
| REST U-87 MG | 299 | 0.442280058 | 1.717368 | 3.51E-06 |
| EZH2 skeletal muscle myoblast | 1384 | 0.392720977 | 1.649838 | 2.80E-14 |
| REST MCF-7 | 458 | 0.409808308 | 1.646251 | 2.48E-06 |
| REST ECC-1 | 722 | 0.399496929 | 1.643402 | 3.09E-08 |
| REST HL-60 | 86 | 0.480253964 | 1.612052 | 0.004949 |
| EZH2 keratinocyte | 1188 | 0.355981809 | 1.490053 | 8.96E-08 |
| ZC3H11A MEL | 472 | 0.36674988 | 1.476064 | 0.000194 |
| REST H1-hESC | 1080 | 0.349275057 | 1.458581 | 1.84E-06 |
| REST HepG2 | 698 | 0.349216124 | 1.435156 | 0.000149 |
| SUZ12 H1-hESC | 2036 | 0.338354834 | 1.433129 | 3.33E-09 |
| NFE2 K562 | 221 | 0.366042615 | 1.381432 | 0.015349 |
| SMC3 MEL | 1333 | 0.318029028 | 1.335158 | 0.000164 |
| SUZ12 K562 | 1410 | 0.317351938 | 1.333961 | 8.48E-05 |
| CBX2 K562 | 1229 | 0.318189963 | 1.333082 | 0.000209 |
| SMC3 SK-N-SH | 1378 | 0.314335308 | 1.32075 | 9.65E-05 |
| RAD21 HCT116 | 1320 | 0.312354013 | 1.310732 | 0.000485 |
| ZC3H11A CH12.LX | 980 | 0.314415669 | 1.309118 | 0.001999 |
| REST GM12878 | 781 | 0.307407737 | 1.268453 | 0.008726 |
| EZH2 myotube | 1139 | 0.303289326 | 1.268067 | 0.002801 |
| CBX8 K562 | 1220 | 0.302817213 | 1.268047 | 0.002275 |
| CTCF ECC-1 | 833 | 0.303850429 | 1.256981 | 0.010153 |
| EZH2 astrocyte | 1431 | 0.296450047 | 1.246711 | 0.002553 |
| ZC3H11A ES-E14 | 624 | 0.3021899 | 1.23455 | 0.025627 |
| REST HeLa-S3 | 816 | 0.292933634 | 1.21122 | 0.028574 |
| CTCF G1E | 1548 | 0.278245596 | 1.172055 | 0.022582 |
| EZH2 fibroblast of dermis | 1446 | 0.278549353 | 1.171854 | 0.025709 |
| RAD21 MCF-7 | 1741 | 0.273076594 | 1.152855 | 0.028444 |

**Table S5. Clinical information of the meningioma patients used for organoid generation.**

| ID | Sex | Age  (yrs) | Location | WHO  grade | Histotype | Ki-67 |
| --- | --- | --- | --- | --- | --- | --- |
| MO1 | M | 49 | Frontal | 1 | Transitional | 2-4% |
| MO2 | M | 50 | Sphenoid Wing | 2 | Atypical | 10-12% |
| MO3 | M | 63 | Parietal | 3 | Anaplastic | 40% |

MO, meningioma organoid.

**Supplementary Figures**

**Figure S1** Verification of primary meningioma cells by Vimentin and EMA staining.


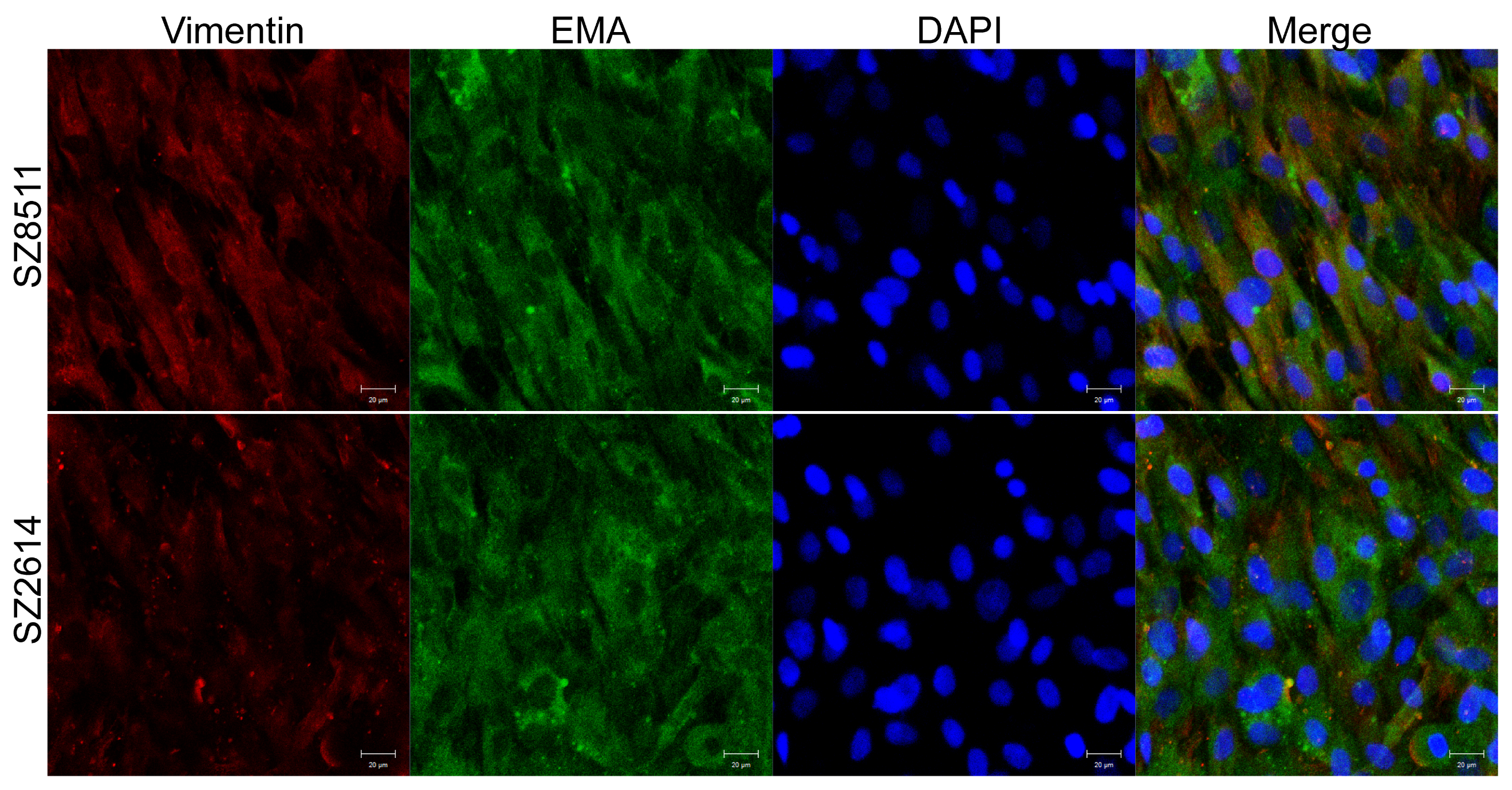


**Figure S2** The effect of CRISPR knockout of SLC7A1, SLC7A2, and SLC7A3 in pan-cancer from the DEPMAP portal.

A lower score means that a gene is more likely to be dependent in a given cell line. A score of 0 is equivalent to a gene that is not essential.


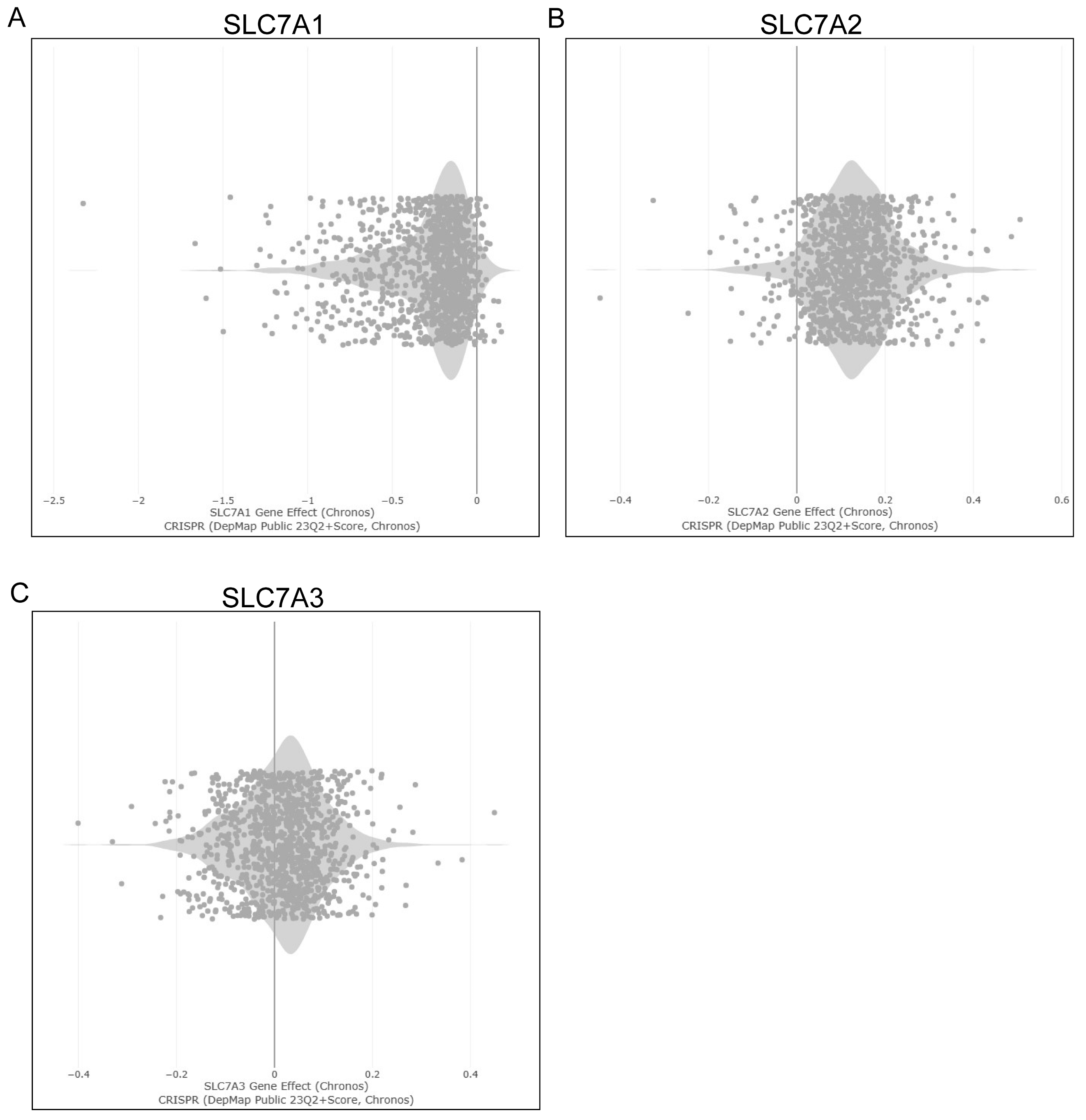


**Figure S3** GSEA revealing the biological processes associated with SLC7A1 expression at the bulk level.

Virtual knockdown analysis using the RNA sequencing data from the GSE136661 dataset was performed to infer the functional role of SLC7A1 in meningioma at the bulk level. GSEA between the high- and low-SLC7A1 groups revealed significant enrichment of cancer hallmark pathways that are closely associated with cell proliferation, such as the G2M checkpoint, E2F targets, and MYC targets.


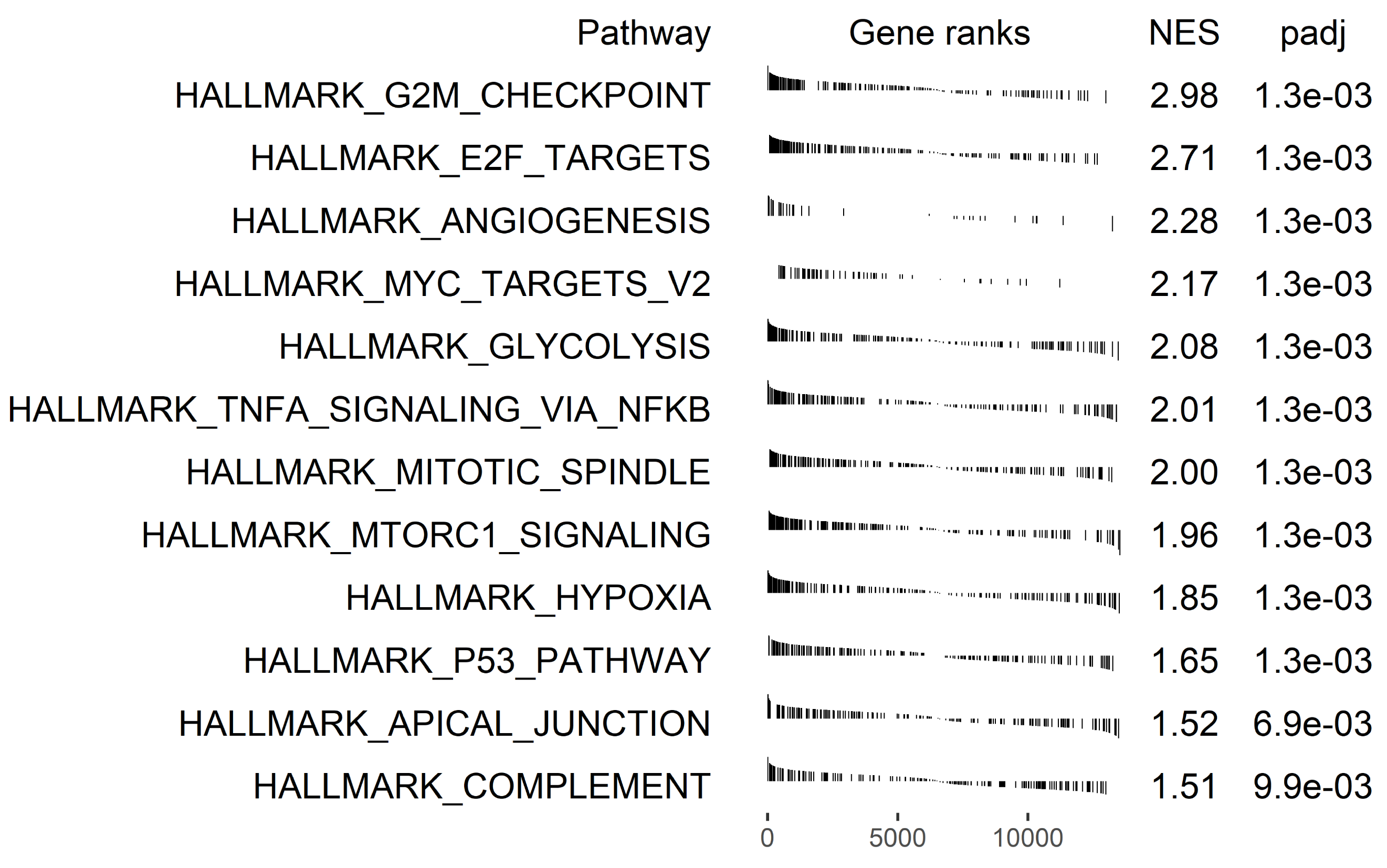


**Figure S4** E2F4 knockdown inhibited the proliferation of meningioma cells. (A and B) Knockdown efficiency of si-E2F4 in IOMM-Lee and SZ8511 verified by RT-qPCR. (C and D) Knockdown efficiency of si-E2F4 in IOMM-Lee and SZ8511 verified by Western blot. (E and F) Effect of E2F4 knockdown on the cell proliferation of IOMM-Lee and SZ8511.


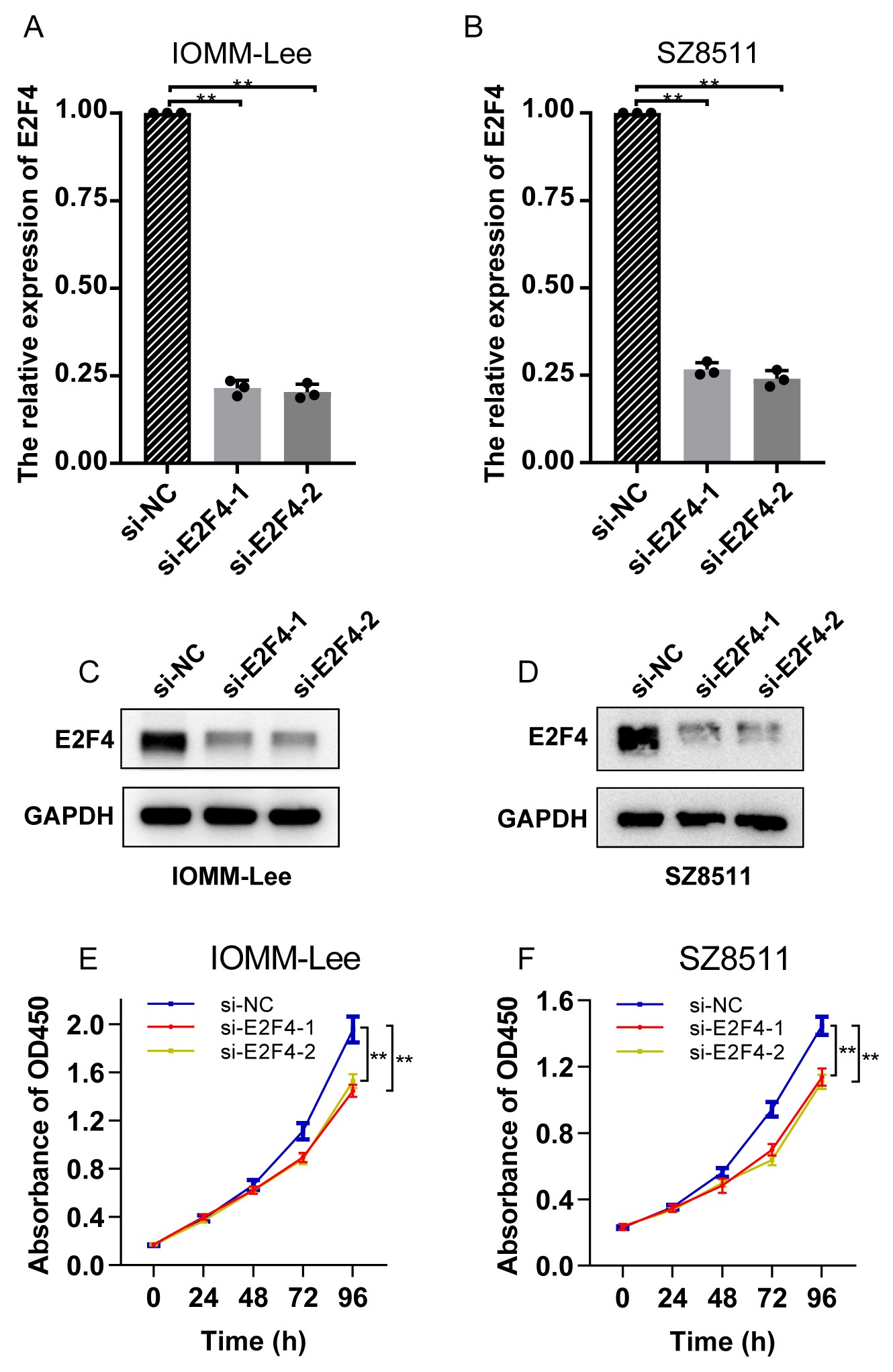


**Figure S5** SLC7A1 knockdown increases the transcriptional activity of REST. (A and B) GSEA revealing an increased transcriptional activity of REST in SLC7A1-knockdown IOMM-Lee and SZ8511. (C) GSVA at the single-cell level also displaying an increased transcription factor activity scores in low-SLC7A1 meningioma cell clusters.


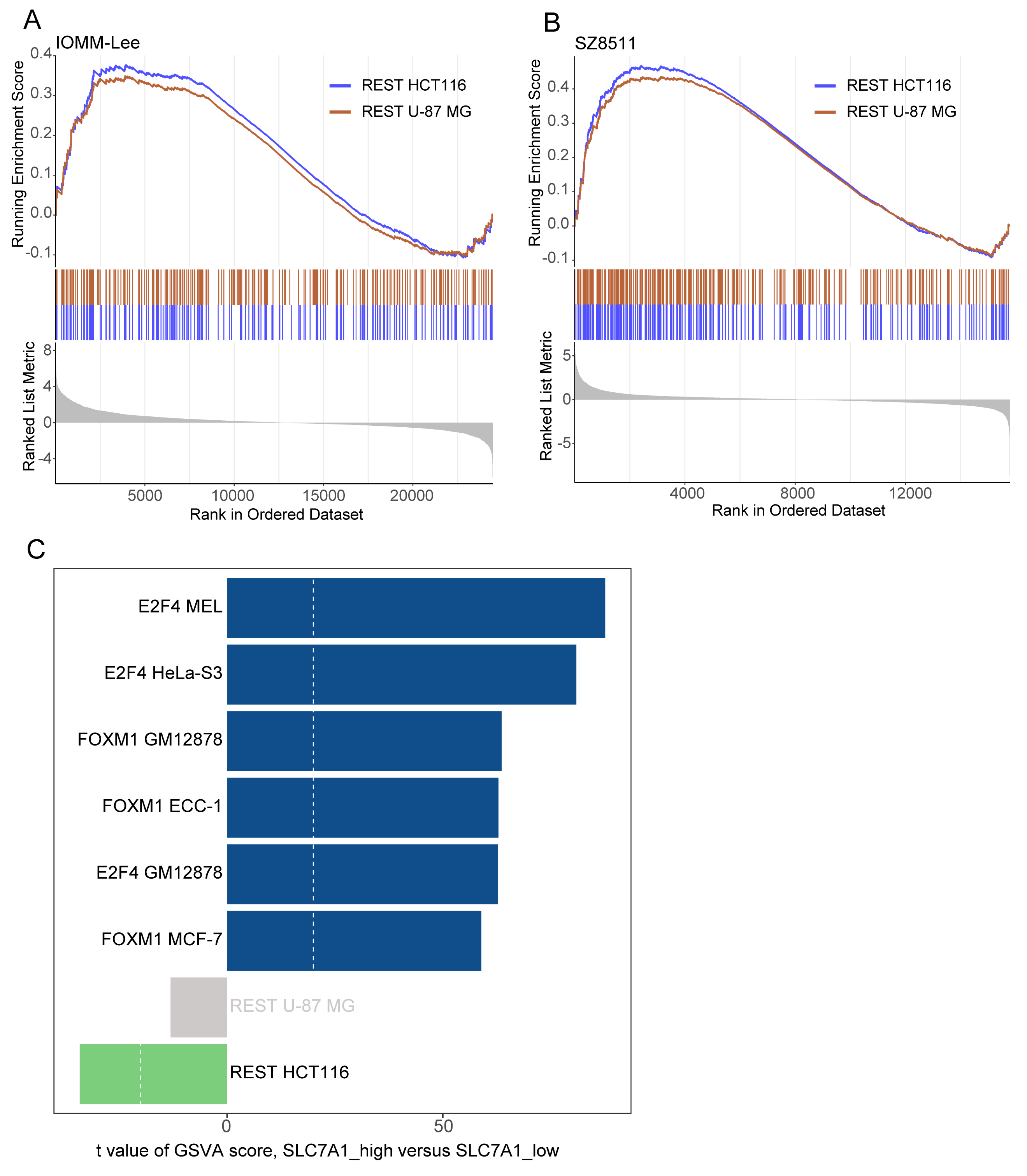


**Figure S6** Evaluation of the proliferation-inhibitory effect of AZ628 on normal dura mater cells. (A, B) Bright‑field micrographs of normal dura mater cells. Primary dura mater cells were isolated from two independent patients and cultured for 2–3 weeks; during this period the cells gradually migrated out from the original tissue fragments. Red arrows indicate normal dura mater tissue. (C) The proliferation inhibition rate of AZ628 on normal dura mater cells at 72 hours, as measured by the CCK‑8 assay. The 72-hour IC50 values for AZ628 are approximately 35.8 μM and 44.1 μM for dura mater cell-1 and dura mater cell-2, respectively.

**
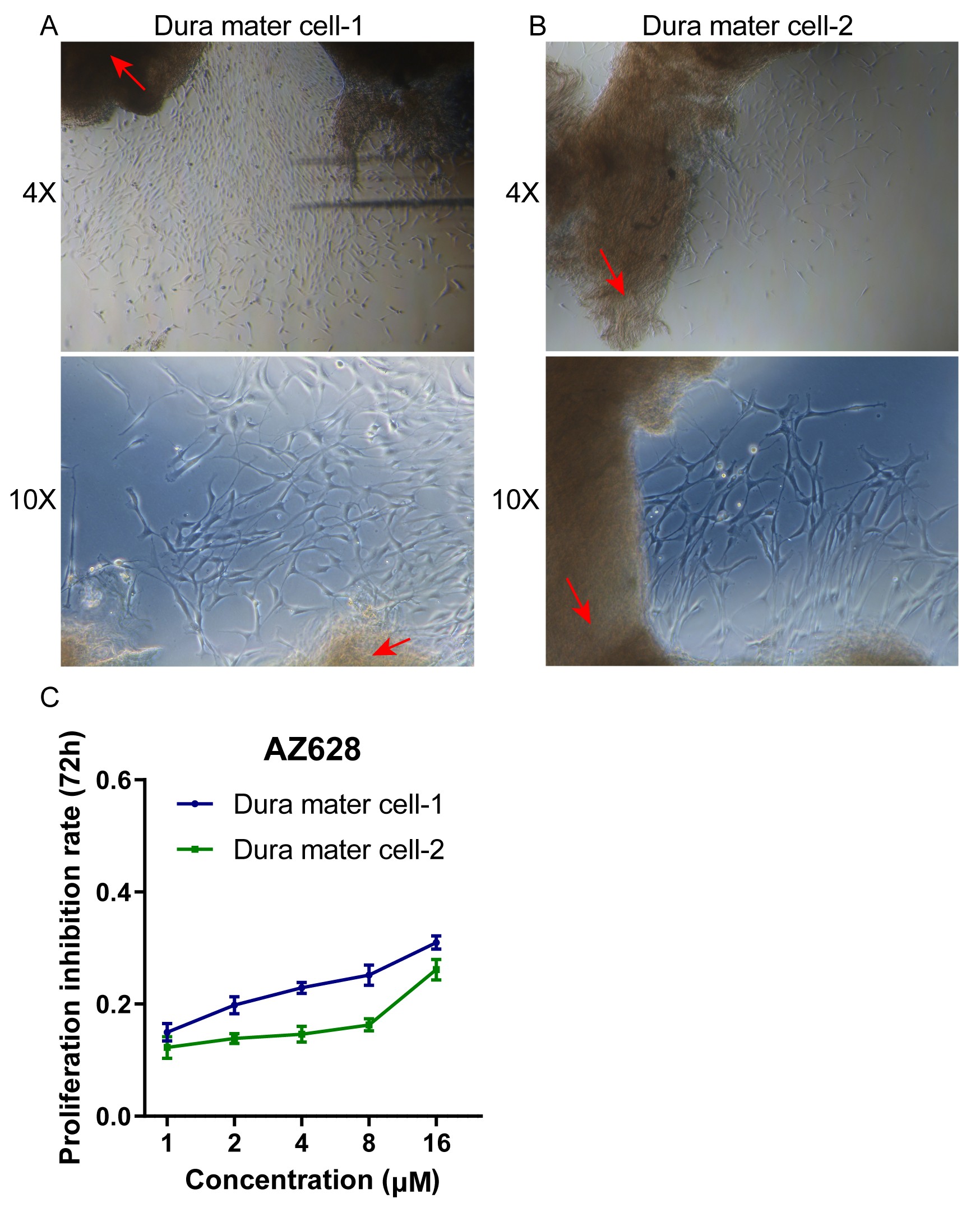
**

**Figure S7** Representative HE staining pictures of the heart, liver, spleen, lung, kidney, and colon of the control and AZ628-treated tumor-bearing mice.


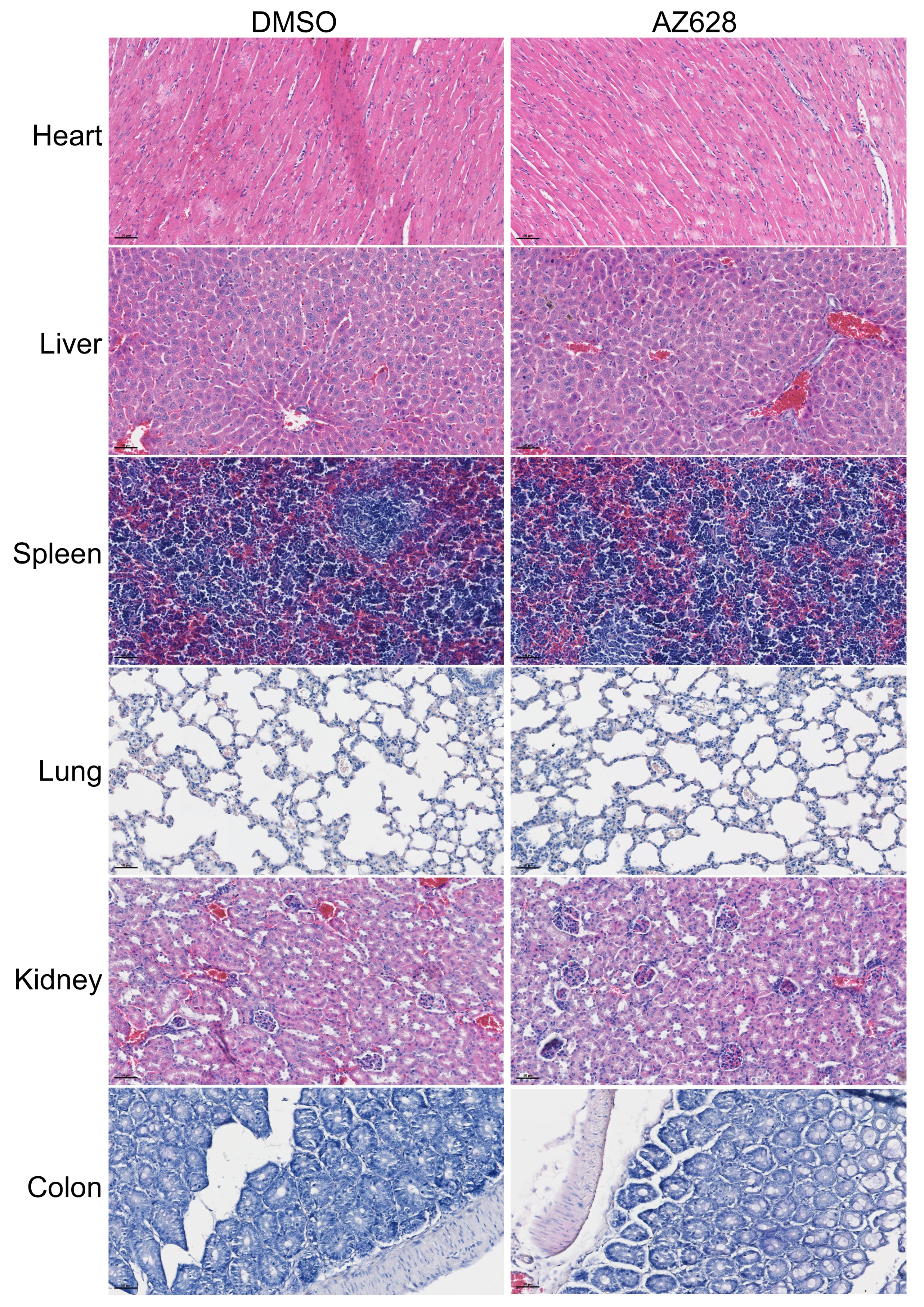


**Figure S8** The effect of AZ628 treatment on the expression of SLC7A1. (A) The effect of AZ628 treatment for 24 hours on SLC7A1 mRNA expression in IOMM-Lee and SZ8511 cells measured by RNA sequencing. (B) The effect of AZ628 treatment for 24 and 48 hours on SLC7A1 mRNA expression in IOMM-Lee and SZ8511 cells measured by RT-qPCR. (C) Western blot displaying the impact of AZ628 treatment on the protein level of SLC7A1 in IOMM-Lee and SZ8511. ** P < 0.01.


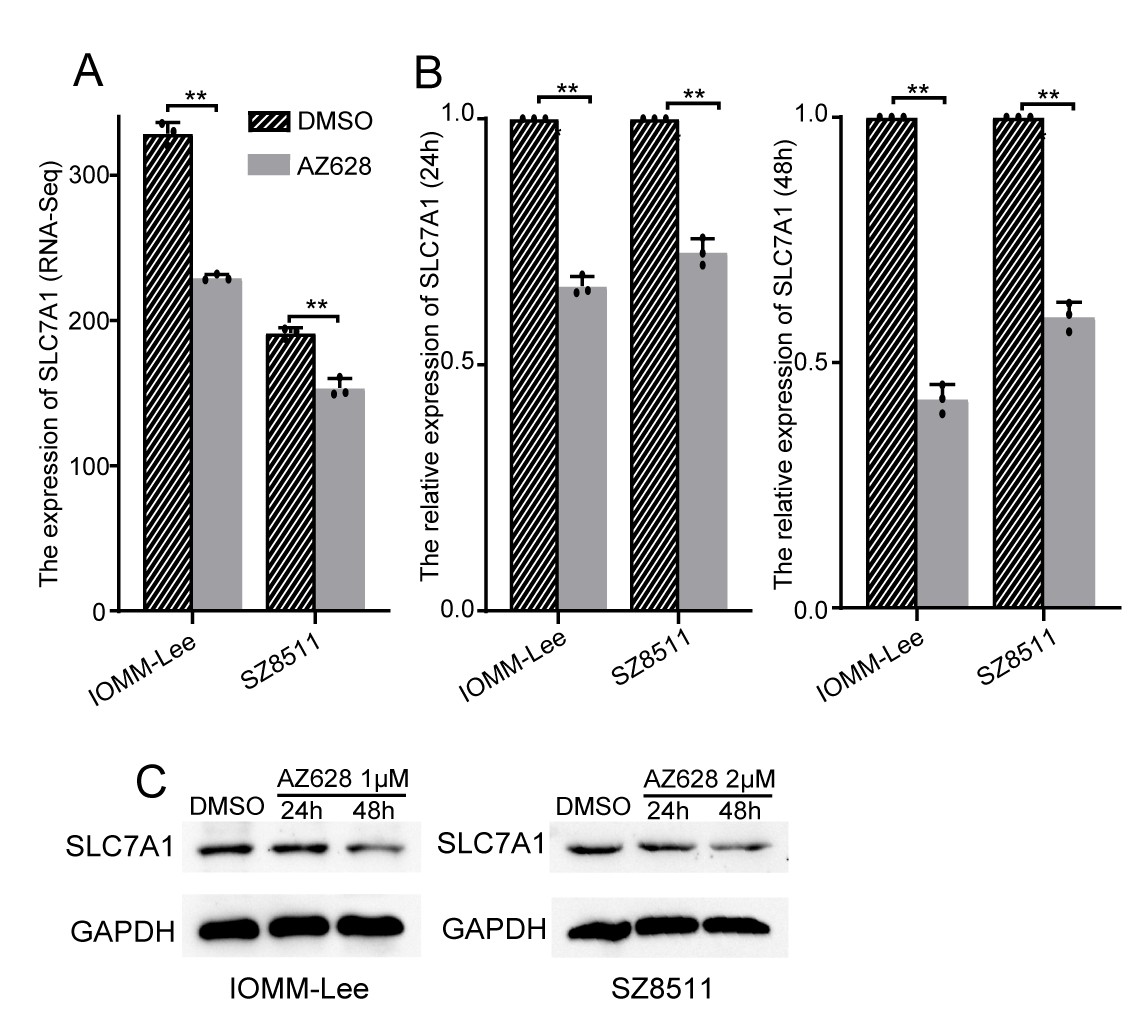

Supplement: Supplementary file 1 — Supplementary Tables and Figures [file 41420_2025_2783_MOESM1_ESM.docx]
