# Supplementary figures and images for: Identification of SLC7A1 as a potential therapeutic target for high-grade meningioma
Source: Cell Death Discov. 2025 Nov 3;11:498. doi: 10.1038/s41420-025-02783-4 (PMC12583633; doi:10.1038/s41420-025-02783-4)

**Figure 3H**

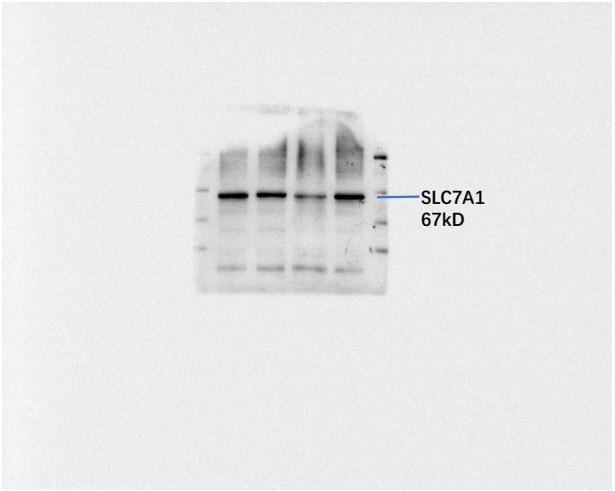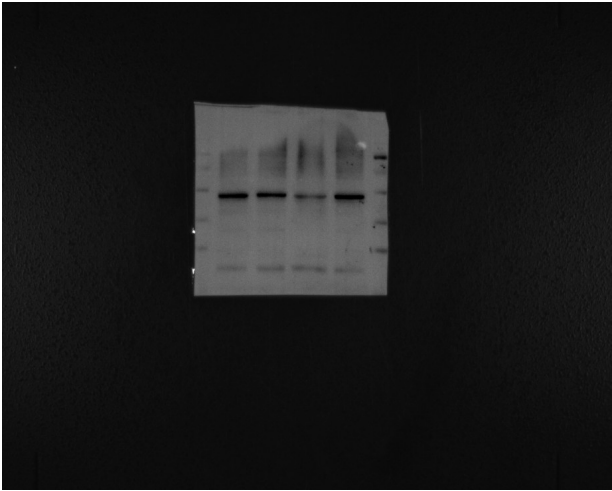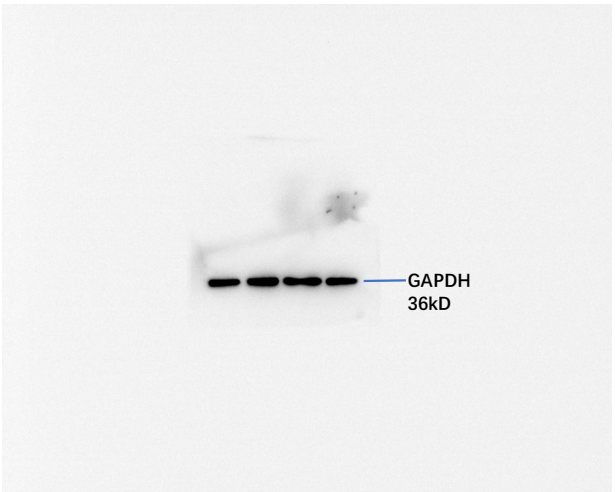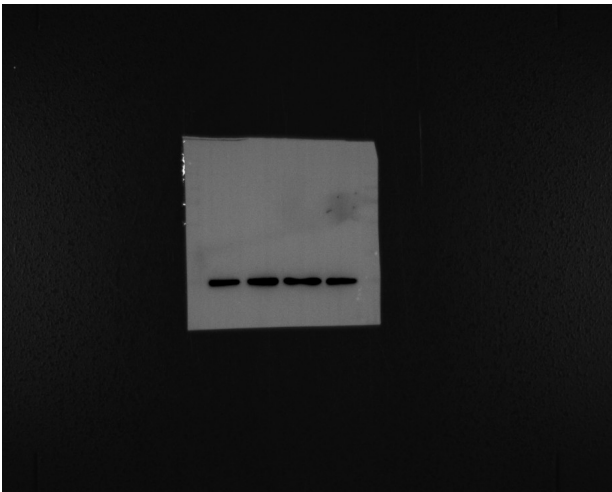

Figure 4J,K

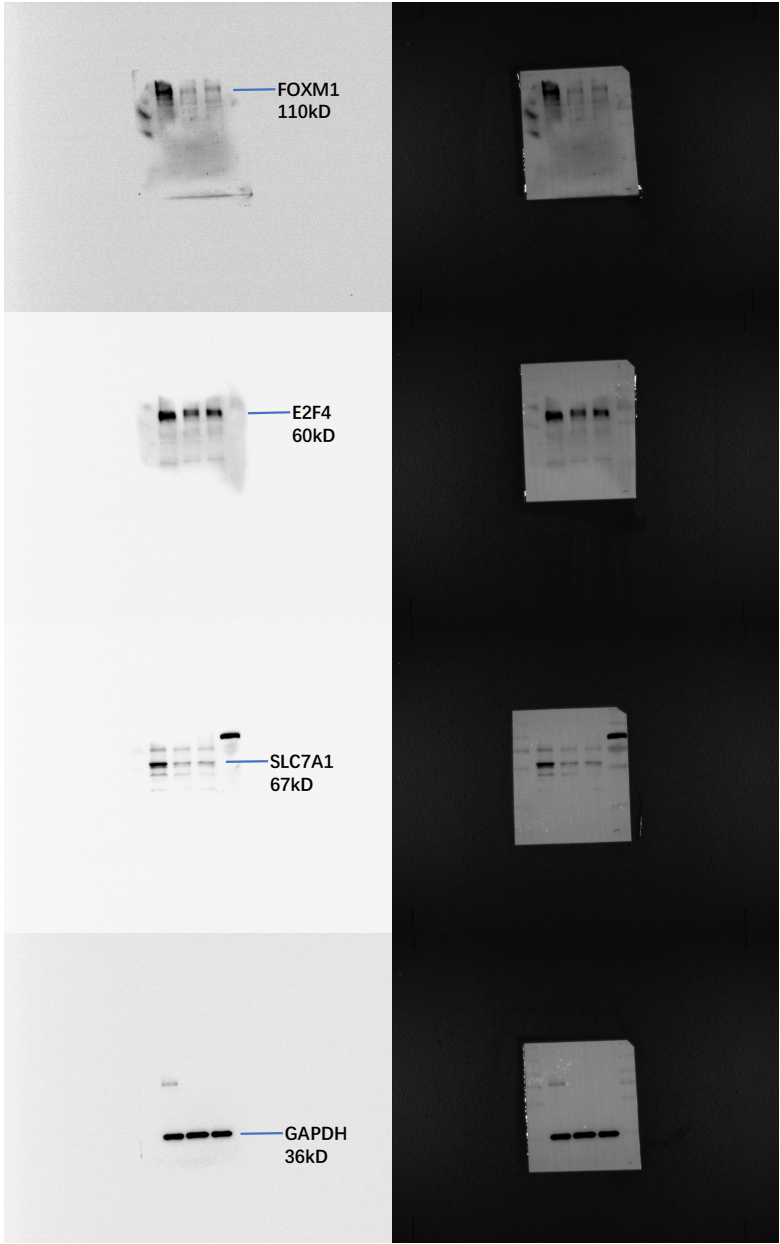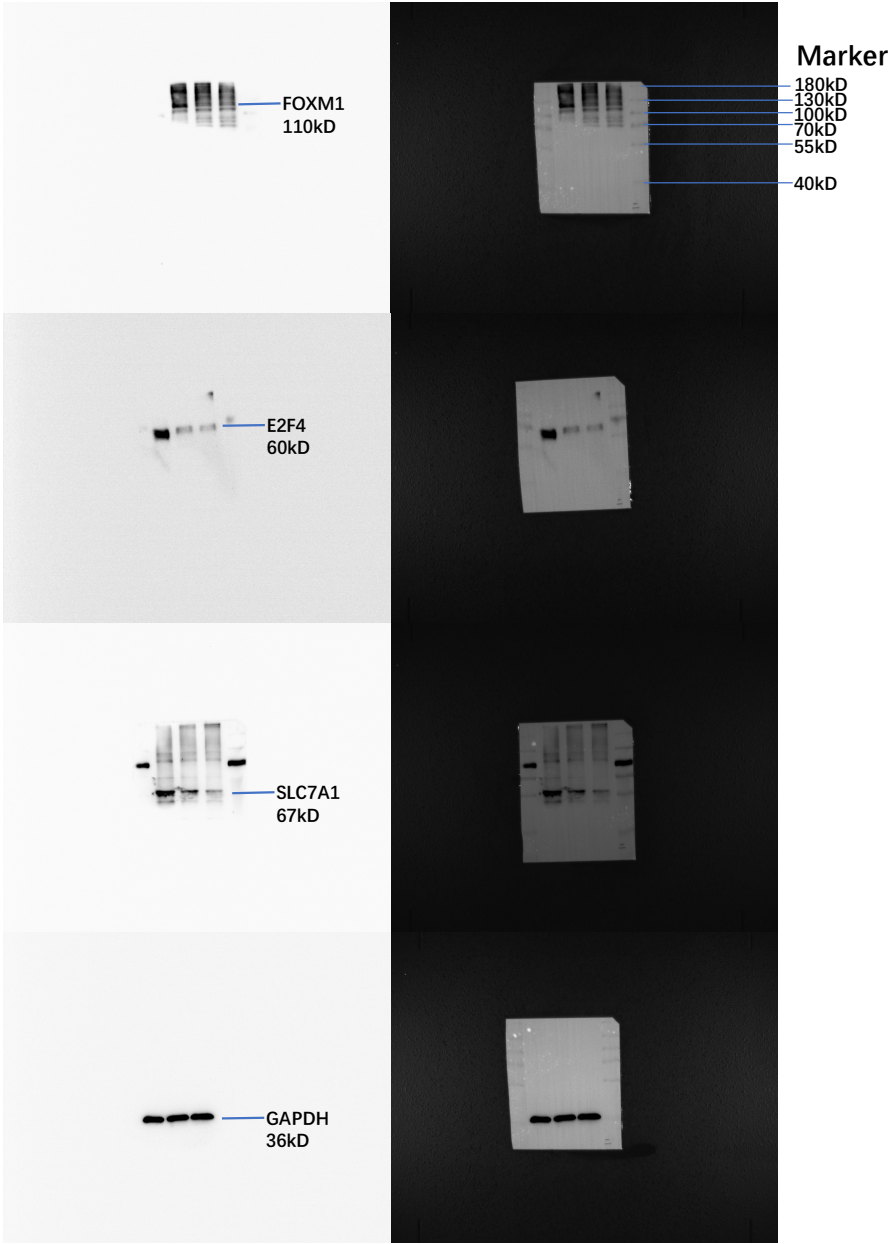

**Figure 5G**

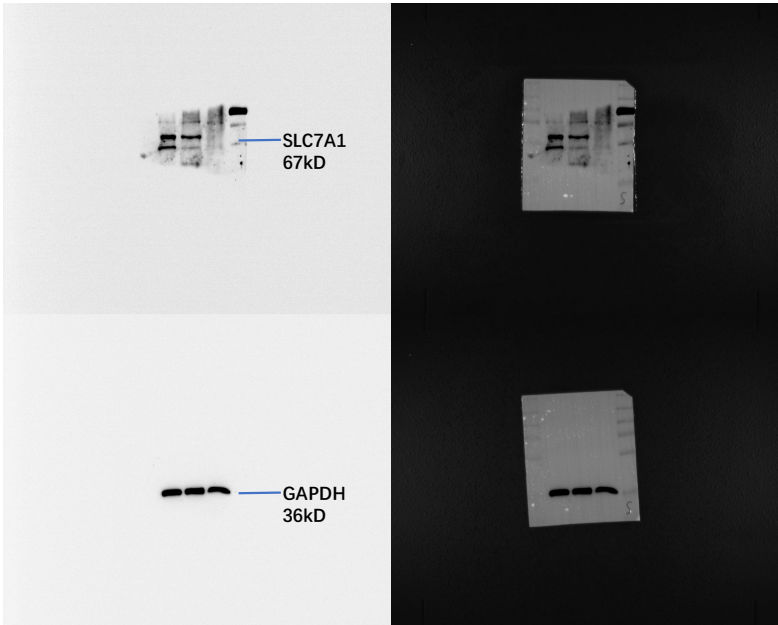

Figure 7G,H

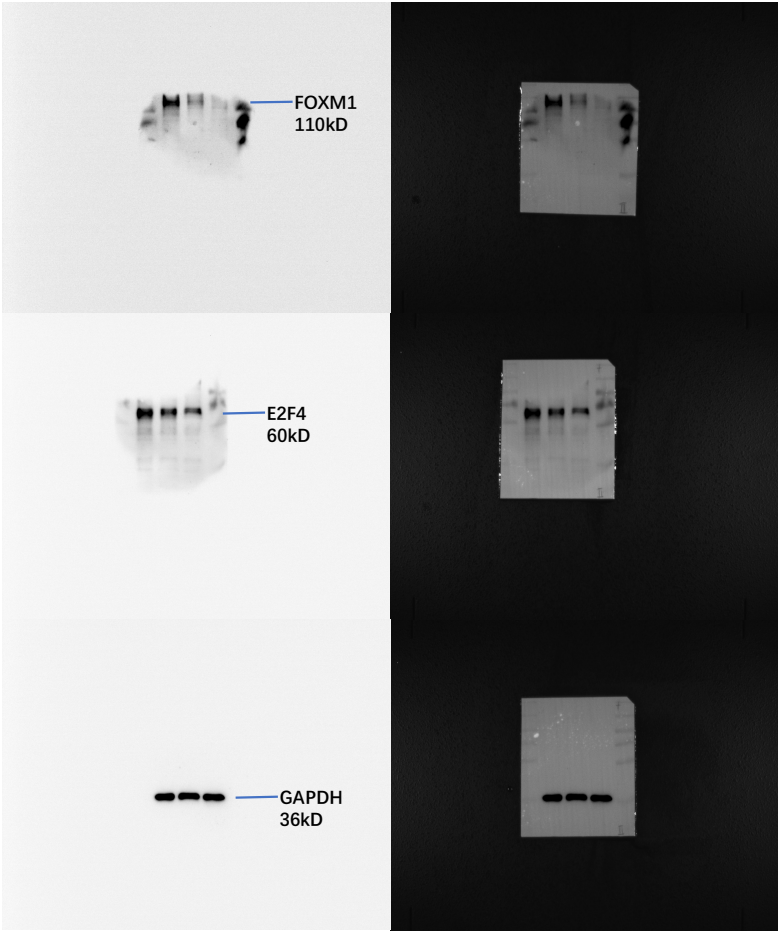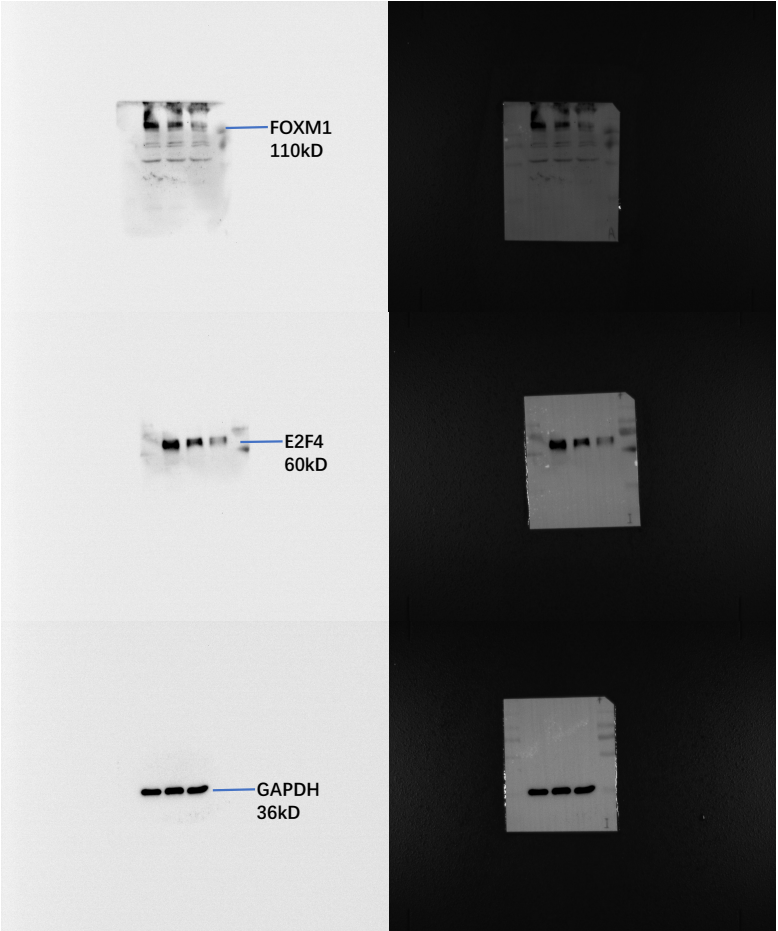

Supplementary Figure S4C,D

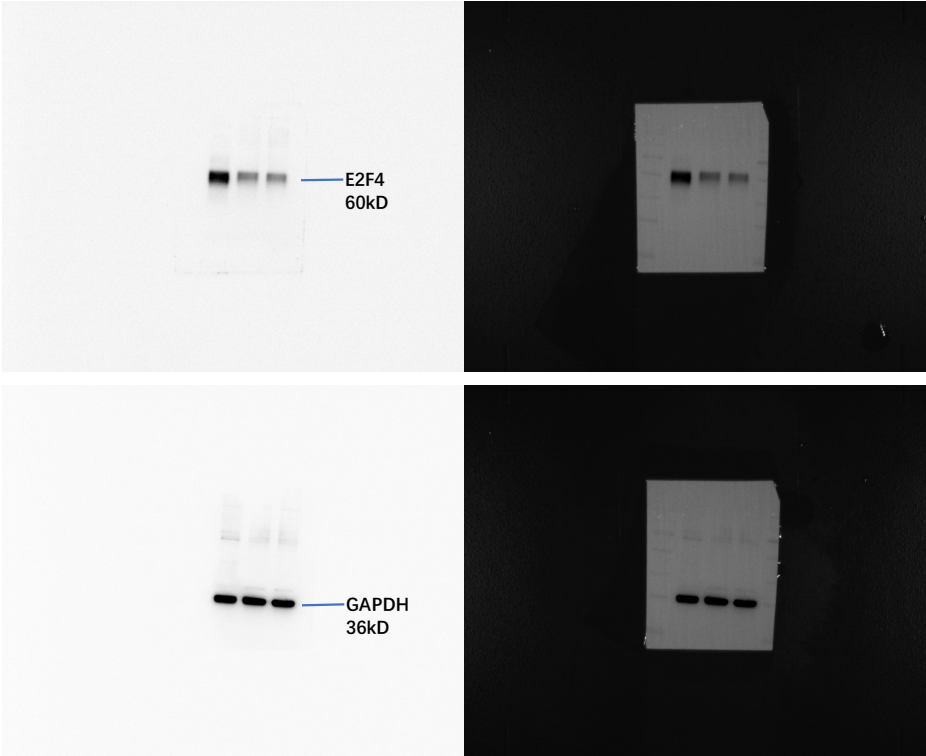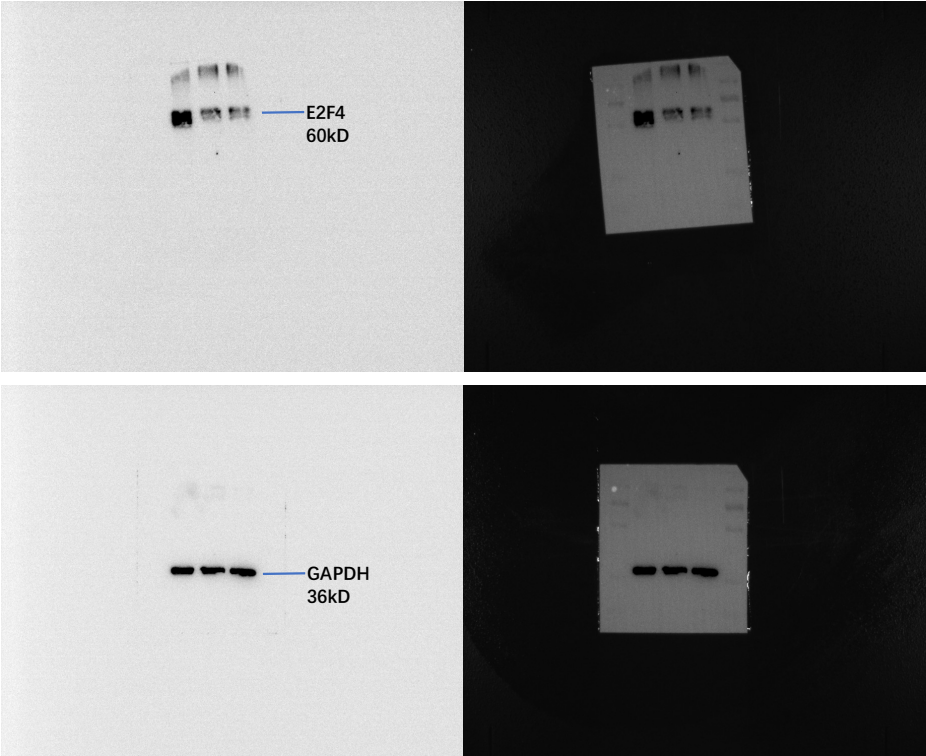

Supplementary Figure S8C

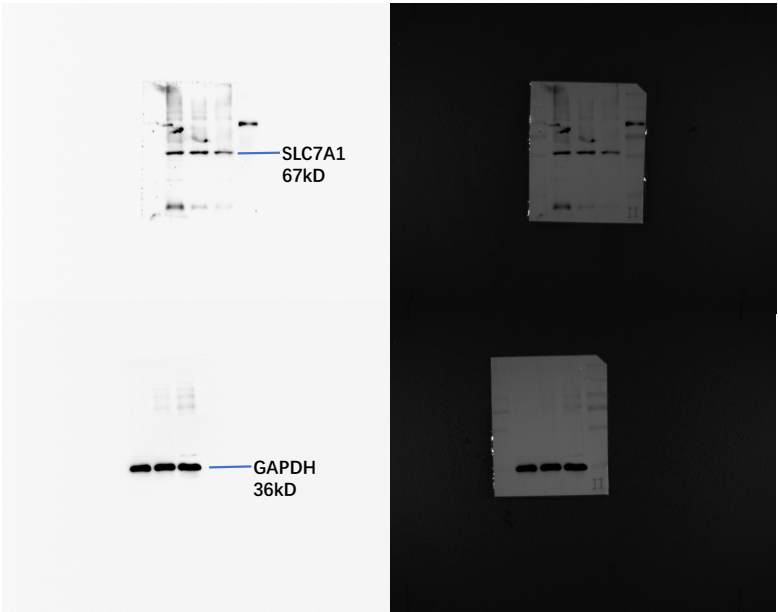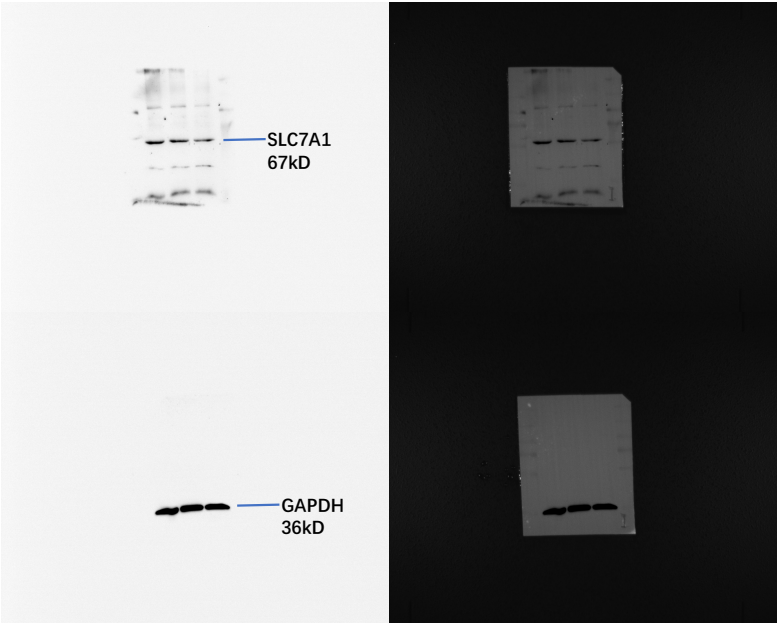

Supplement: Supplementary file 2 — un-cropped images of the original western blots [file 41420_2025_2783_MOESM2_ESM.pdf]
